# Time-Dependent Strategies in Repeated Asymmetric Public Goods Games

**DOI:** 10.1007/s13235-025-00627-5

**Published:** 2025-02-07

**Authors:** Valentin Hübner, Christian Hilbe, Manuel Staab, Maria Kleshnina, Krishnendu Chatterjee

**Affiliations:** 1 Institute of Science and Technology Austria, 3400 Klosterneuburg, Austria; 2 Max Planck Research Group Dynamics of Social Behavior, Max Planck Institute for Evolutionary Biology, 24306 Plön, Germany; 3 School of Mathematical Sciences, Queensland University of Technology, Brisbane, QLD 4000 Australia; 4 School of Economics, The University of Queensland, Brisbane, QLD 4067 Australia

**Keywords:** Social dilemmas, Public goods games, Inequality, Direct reciprocity, 91A05, 91A06, 91A10, 91A20

## Abstract

The public goods game is among the most studied metaphors of cooperation in groups. In this game, individuals can use their endowments to make contributions towards a good that benefits everyone. Each individual, however, is tempted to free-ride on the contributions of others. Herein, we study repeated public goods games among asymmetric players. Previous work has explored to which extent asymmetry allows for full cooperation, such that players contribute their full endowment each round. However, by design that work focusses on equilibria where individuals make the same contribution each round. Instead, here we consider players whose contributions along the equilibrium path can change from one round to the next. We do so for three different models – one without any budget constraints, one with endowment constraints, and one in which individuals can save their current endowment to be used in subsequent rounds. In each case, we explore two key quantities: the welfare and the resource efficiency that can be achieved in equilibrium. Welfare corresponds to the sum of all players’ payoffs. Resource efficiency relates this welfare to the total contributions made by the players. Compared to constant contribution sequences, we find that time-dependent contributions can improve resource efficiency across all three models. Moreover, they can improve the players’ welfare in the model with savings.

## Introduction

Cooperation is typically conceptualised as a behaviour that is costly for the individual but beneficial to the group [39]. Examples of cooperation abound, ranging from small favours among friends to collective efforts to mitigate climate change. These cooperative interactions can, and have been, described with game theory [8, 31]. This literature has produced rich predictions about potential mechanisms that can sustain cooperation [27, 34]. One such mechanism is direct reciprocity [5, 7, 40, 44]. Here, individuals are assumed to engage in the same interaction repeatedly, over many rounds. Repeated interactions allow players to condition their behaviour on the previous history of play. In this way, they can enforce mutual cooperation despite any short-run temptations to free-ride [13, 14].

Traditionally, many models of direct reciprocity, especially in the evolutionary game theory literature, assume that interactions are symmetric [4, 12, 16, 19, 22, 25, 26, 30, 35, 37, 38, 41, 42, 46]. This means that players are completely interchangeable with respect to their actions and feasible payoffs. More recently, however, the evolution of cooperation among asymmetric players has received more attention [1–3, 9, 11, 24, 29, 32, 33, 45]. This interest has also been spurred by empirical studies that explore the role of inequality in controlled experiments [6, 10, 17, 21, 28, 36, 43, 47].

Oftentimes, these studies are based on some variation of the linear public goods game. In this game, players obtain their fixed endowments in the beginning of each round. Then they independently decide how much of their endowment they wish to contribute to the public good. Contributions are multiplied by some productivity factor, and the resulting amount is evenly split among all group members. There are various ways to allow for asymmetry in this game. For example, players may have unequal endowments, unequal productivities, or both. The main takeaway from the above-mentioned studies is that endowment inequality tends to be detrimental to cooperation. However, as shown by [20] and [23], there can be exceptions. If individuals already differ in their productivity, it can become easier to sustain full cooperation if they also differ in their endowments. As a rule of thumb, a player’s endowment ought to be larger the more productive that player is.

However, the studies of [20] and [23] consider a rather restricted question. They ask: Under which conditions are there subgame perfect equilibria in which all players contribute their full endowment in every round? In particular, they thereby only consider equilibria whose resulting contribution sequence along the equilibrium path is constant. Instead, in the following we are interested in contribution sequences that can vary in time. We ask: Once individuals have the ability to make time-dependent contributions along the equilibrium path, to which extent can they achieve outcomes that are infeasible with constant contribution sequences?

To this end, we study three different but related models of public good provision. The first is most convenient from a mathematical perspective. Here, players can make arbitrary (non-negative) contributions each round. In particular, contributions are not constrained by any endowments that individuals might have in that round. Instead, we only require that the players’ overall discounted contributions over the entire game are bounded (with the upper bound being arbitrary). We refer to this model as the ‘base model’. The second model reproduces typical public goods game models, such as the ones considered in [20] and [23]. Here, a player’s contribution each round is bounded by the player’s assigned endowment. Accordingly, we speak of the ‘endowment model’. Finally, the third model is a hybrid of the first two. Here, players obtain a fixed and constant endowment each round. But now they can decide to deposit some of this endowment into a savings account, which is then available in the next round. Under this assumption, contributions each round are not bounded by the players’ endowments anymore. Instead, they are bounded by the players’ accumulated endowments up to that point. We call this the ‘savings model’.

For all three models, we consider the equilibrium outcomes that can be achieved with time-dependent contributions. We compare them to the possible equilibrium outcomes when players are required to make a fixed and constant contribution along the equilibrium path. We make this comparison based on two key quantities. One quantity is the group’s welfare in equilibrium (the total sum of the players’ payoffs). As we discuss in more detail below, this quantity is particularly relevant in the endowment model and in the savings model. The other quantity is an equilibrium’s resource efficiency (the ratio of the group’s welfare relative to the players’ total contributions). This quantity is relevant for all three models.

We characterise under which condition time-dependent contributions allow for equilibria with larger resource efficiency (compared to equilibria based on constant contributions). We do so for arbitrary discount factors. In particular, we do not require that players are sufficiently patient, as often done in the classical folk theorem literature [13, 14]. Our results depend on the group size and on the number of players with the highest productivity. In particular, we find that when there is a unique player with maximum productivity, time-dependent contributions provide an advantage. With respect to welfare maximisation, we find a similar result – but only for the savings model.

## The Base Model

### Model Setup

**Fig. 1:**
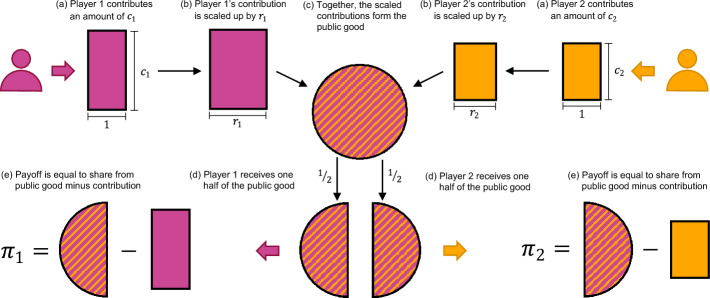
A one-round asymmetric public goods game. To illustrate the base model, we consider $n\!=\!2$ players. They freely choose the size of their contributions, $c_1$ and $c_2$. Contributions are enhanced by the individual productivity factors, which are $r_1 \!=\! 1.5$ and $r_2 \!=\! 1.1$ in this example. The size of the public good is the sum of all enhanced contributions, ${\textbf{r}}^\intercal {\textbf{c}}\!=\!r_1c_1\!+\!r_2c_2$. Each player receives an equal share of this sum. The payoff of each player then equals their share of the public good minus their contribution

We start with the base model (as illustrated in Fig. 1), which we will use to derive our first results. These results will also have important implications for the other two models studied subsequently.

In the base model, a group of $n\! \ge \! 2$ players interacts for an indefinite sequence of rounds. In every round *t*, each player *i* decides which non-negative amount $c_i(t)$ to contribute towards the public good. There is no limit on how much players may contribute. Hence, $c_i(t) \!\in \! {\mathbb {R}}_{\ge 0}$. These individual contributions can be collected in a vector, ${\textbf{c}}(t) \!=\! (c_1(t), \dots , c_n(t))^\intercal$. We refer to a sequence $\big (\textbf{c}(t)\big )_t$ of contribution vectors as a contribution sequence, or as a ‘play’ of the game. If ${\textbf{c}}(t) \!=\! {\textbf{c}}(0)$ for all times *t*, the contribution sequence is called constant. Otherwise, it is time-dependent.

Contributions of each player *i* are multiplied by their productivity factor $r_i$ and added to the public good. The total public good is then evenly shared among all players. This is a slight generalisation of the standard formulation of the public goods game, according to which every player has the same productivity. In the following, we use ${\textbf{r}} \!=\! (r_1, \dots , r_n)^\intercal$ to denote the vector of all productivities. Based on this notation, we can write payer *i*’s payoff $\pi _i(t)$ in round *t* as

1 $$\begin{aligned} \pi _i(t) = \frac{1}{n} \textbf{r}^\intercal {\textbf{c}}(t) - c_i(t). \end{aligned}$$

We assume productivities satisfy $1 \!<\! r_i \!<\! n$ for all players *i*. The first inequality $r_i \!>\! 1$ ensures that the group’s total payoff (across all members) is increasing in each player’s contributions. The second inequality, $r_i \!<\! n$, on the other hand, ensures that each individual is tempted to give as little as possible. Together, these two inequalities render the game a social dilemma. For each player, there is a conflict between their private interest and the collective interest of the group.

To define the players’ payoffs over the entire repeated game, we assume players value each subsequent round at a discount of $\delta$, with $0\!<\! \delta \!<\! 1$. Accordingly, when the contribution sequence $\big ({\textbf{c}}(t)\big )_t$ is bounded, we define the total payoff of each player as the weighted sum of their payoffs each round,

$$\begin{aligned} \hat{\pi }_i = (1\!-\!\delta ) \sum _{t=0}^\infty \delta ^t \pi _i(t). \end{aligned}$$

Here, the term $1-\delta$ serves as a normalising factor. It ensures that repeated-game payoffs are comparable to the game’s one-shot payoffs. Because we assumed the contribution sequence to be bounded, the above sum is guaranteed to converge. We do not define a total payoff for unbounded contribution sequences.

In a common alternative interpretation of repeated games with discounting, which is applicable to our base model and model variation I, but not model variation II, the number of rounds is finite and random, with $\delta$ being not a discount factor but the round-wise continuation probability. This means that after each round, with probability $\delta$ the game continues for at least one more round and with probability $1-\delta$ it ends. In that interpretation, all rounds have equal value given that they are played, and the expected number of rounds is $1/(1-\delta )$. Intuitively, this suggests that higher values of $\delta$, which mean longer games, are conducive to cooperation.

#### Notation 1

In this base model, a game is fully specified by the players’ productivities ${\textbf{r}}$ and by the discount factor $\delta$. We denote the corresponding game as $\Gamma _{\!\textrm{B}}({\textbf{r}}, \delta )$.

For our subsequent analysis, it will be useful to consider the weighted sum of a player’s contributions after a given time *t*. Formally, these continuation contributions of player *i* are defined as

2 $$\begin{aligned} \bar{c}_i(t) = (1-\delta ) \sum _{\tau =0}^\infty \delta ^\tau c_i(t+\tau ). \end{aligned}$$

One can also define a sequence that collects the respective continuation contributions for each round, $\big (\bar{\textbf{c}}(t)\big )_t = \big (\bar{{\textbf{c}}}(0), \bar{{\textbf{c}}}(1), \bar{{\textbf{c}}}(2), \dots \big )$. We call $\big (\bar{{\textbf{c}}}(t)\big )_t$ the continuation contribution sequence associated with contribution sequence $(\textbf{c}(t))_t$. Every contribution sequence uniquely specifies a continuation contribution sequence and vice versa. Analogously, we can also define continuation payoffs at time *t*,

$$\begin{aligned} \bar{\pi }_i(t) = (1-\delta ) \sum _{\tau =0}^\infty \delta ^\tau \pi _i(t+\tau ). \end{aligned}$$

We write $\bar{\varvec{\uppi }}(t)\!=\!\big ({\bar{\pi }}_1(t),\ldots ,{\bar{\pi }}_n(t)\big )$ for the respective vector, and note that $\hat{\varvec{\uppi }} \!=\! \bar{\varvec{\uppi }}(0)$. Similarly, we write $\hat{{\textbf{c}}} = \bar{\textbf{c}}(0)$ for the continuation contributions at time zero. We call $\hat{{\textbf{c}}}$ the total contribution vector. By linearity of the one-round payoffs (1), we have

3 $$\begin{aligned} \hat{\pi }_i = \frac{1}{n} {\textbf{r}}^\intercal \hat{{\textbf{c}}} - \hat{c}_i. \end{aligned}$$

That is, each player’s total payoff is uniquely determined by the total contribution vector. By definition, this payoff $\hat{\pi }_i$ is the quantity that player *i* aims to maximise.

Players make their decisions based on their strategies. A strategy $\sigma _i$ for player *i* is a function that assigns to each initial contribution sequence $\big ({\textbf{c}}(0), {\textbf{c}}(1), \dots , {\textbf{c}}(t-1)\big )$ a next contribution value $c_i(t) \!=\! \sigma _i\big (({\textbf{c}}(\tau ))_{\tau < t}\big ) \!\in \! {\mathbb {R}}_{\ge 0}$. A strategy is called bounded if it always produces a bounded contribution sequence (irrespective of the co-players’ strategies). An assignment of one strategy to each player $(\sigma _i)_i$ is called a strategy profile. A strategy profile is bounded if all its strategies are bounded.

### Sustainable Contribution Sequences

In the following, we are particularly interested in those strategy profiles that form a subgame perfect equilibrium [SPE, or ‘equilibrium’, see 15]. We say a bounded strategy profile is in equilibrium when no player has an incentive to deviate, after no finite sequence of moves. Formally, $(\sigma _i)_i$ is in equilibrium if there is no initial contribution sequence ${\textbf{c}}(0), \dots , {\textbf{c}}(t-1)$ such that some player *j* could get a larger payoff by deviating towards another bounded strategy $\sigma _j^*$ after that time *t*. For a given game $\Gamma _{\!\textrm{B}}({\textbf{r}}, \delta )$, we call a contribution sequence sustainable if it is the contribution sequence of some equilibrium strategy profile. A total contribution vector  $\hat{{\textbf{c}}}$ is sustainable if it is the total contribution vector of a sustainable contribution sequence .

To derive our main results, we make extensive use of the previously published Theorem 1 below. This theorem gives us a comfortable characterisation of sustainable contribution sequences .

#### Theorem 1

( [23]) For a given game $\Gamma _{\!\textrm{B}}({\textbf{r}}, \delta )$, define an associated $n\!\times \!n$ matrix $D=(D_{ij})$, called the productivity matrix in zero-diagonal form, by

4 $$\begin{aligned} D_{ij} = \left\{ \begin{array}{ll} r_j/(n\!-\!r_i) & \quad \text { if}~i\ne j\\ 0 & \quad \text { if}~i=j. \end{array} \right. \end{aligned}$$

Then a contribution sequence $({\textbf{c}}(t))_t$ is sustainable if and only if the associated continuation contributions satisfy

5 $$\begin{aligned} \bar{{\textbf{c}}}(t) \le \delta D \bar{{\textbf{c}}}(t+1) \quad \text { for all}~t. \end{aligned}$$

With Theorem 1, we can determine whether a given contribution sequence is sustainable by checking if the associated continuation contribution sequence $(\bar{{\textbf{c}}} (t))_t$, as defined by (2), satisfies (5) for all *t*. The two following corollaries are immediate consequences of this theorem.

#### Corollary 2

For any game $\Gamma _{\!\textrm{B}}({\textbf{r}}, \delta )$, the set of sustainable contribution sequences $({\textbf{c}}(t))_t$, the set of sustainable continuation contribution sequences $(\bar{\textbf{c}}(t))_t$, and the set of sustainable total contribution vectors $\hat{{\textbf{c}}}$ are closed under addition and multiplication by a non-negative scalar (that is, they are convex cones).

This convexity result implies that whether or not a contribution sequence is sustainable only depends on the relative magnitude of the players’ contributions. This result holds because payoffs depend linearly on contributions. Therefore, scaling all contributions up or down by the same positive factor does not affect whether or not the equilibrium conditions are satisfied.

#### Corollary 3

In a given game $\Gamma _{\!\textrm{B}}({\textbf{r}}, \delta )$, a constant contribution sequence of $(\hat{{\textbf{c}}})_t$ is sustainable if and only if

6 $$\begin{aligned} \hat{{\textbf{c}}} \le \delta D \hat{{\textbf{c}}}. \end{aligned}$$

So we have a set of *n* linear constraints that defines the set of feasible constant contribution sequences. We can use Corollary 3 to derive a version of the folk theorem, applied to our setup. The folk theorem famously relates the possible equilibrium payoffs in the repeated game to the properties of the one-shot payoffs [13, 14]. To state our version, we note that in our public goods game, players can always guarantee a non-negative payoff (by not contributing anything). Hence, we say a contribution vector $\hat{{\textbf{c}}}$ for the one-shot game is individually rational if it yields a non-negative payoff to each player.

#### Theorem 4

(Folk theorem of repeated games) Constant contributions $(\hat{\textbf{c}})_t\!\in \!\mathbb {R}_{\ge 0}$ are sustainable in the game $\Gamma _{\!\textrm{B}}({\textbf{r}}, \delta )$ for sufficiently large $\delta$ if and only if $\hat{{\textbf{c}}}$ is individually rational.

The condition for a contribution vector $\hat{{\textbf{c}}}$ to be individually rational can be written as

7 $$\begin{aligned} \max _i \hat{c}_i \le \frac{1}{n} {\textbf{r}}^\intercal \hat{{\textbf{c}}}. \end{aligned}$$

An equivalent formulation of the folk theorem is therefore: the constant contribution sequence $(\hat{{\textbf{c}}})_t$ is sustainable for sufficiently large $\delta$ if and only if Eq. (7) holds.

The above results allow us to characterise the properties of sustainable contribution sequences. Perhaps one of the most important properties is whether or not the contribution sequence entails at least some cooperation. More specifically, we define a play to be non-defective if at least one player makes a positive contribution in at least one round (i.e., $\hat{{\textbf{c}}} \!\ne \! \textbf{0}$). Otherwise we call the play defective. It is easy to see that for non-defection to be sustainable, not just one, but at least two players have to make positive contributions. This is because a hypothetical lone non-defector would benefit from deviating towards full defection. If non-defection is sustainable in a given game $\Gamma _{\!\textrm{B}}({\textbf{r}}, \delta )$, then we say that $\Gamma _{\!\textrm{B}}({\textbf{r}}, \delta )$ allows for non-defection. Any productivity vector ${\textbf{r}}$ (satisfying the general requirement $r_i \!>\! 1$ for all *i*) allows for non-defection when $\delta$ is sufficiently large [20, SupplementaryInformation,Proposition 2].

### Welfare and Resource Efficiency

While the binary distinction between defection and non-defection is useful, not all forms of non-defection are equally desirable. After all, even non-defective contribution sequences might result in payoffs arbitrarily close to the full defection payoff of zero. Therefore, in the following we introduce two other key metrics of interest. The first metric is the (overall) welfare *W* of a given play, which equals the sum of all payoffs,

8 $$\begin{aligned} W = \sum _{i=1}^n {\hat{\pi }}_i. \end{aligned}$$

This welfare can be expressed as a function of the total contribution vector $\hat{{\textbf{c}}}$ as

9 $$\begin{aligned} W(\hat{{\textbf{c}}}) = ({\textbf{r}} - {\textbf{1}})^\intercal \hat{{\textbf{c}}}. \end{aligned}$$

By this equation, non-defective plays have $W \!>\! 0$, whereas defective plays have $W \!=\! 0$.

By Corollary 2, any non-defective contribution sequence can be scaled arbitrarily without affecting their sustainability. It follows that our base model allows for arbitrary welfares. As an alternative metric that is still relevant with unlimited resources, we measure how efficiently the players are able to use them. The resource efficiency *E* of a non-defective play is defined as the sum of all payoffs divided by the sum of all contributions,

10 $$\begin{aligned} E = \frac{\sum _{i=1}^n {\hat{\pi }}_i}{\sum _{i=1}^n {\hat{c}}_i}. \end{aligned}$$

Resource efficiency, too, is a function of the total contribution vector :

11 $$\begin{aligned} E(\hat{{\textbf{c}}}) = \frac{{\textbf{r}}^\intercal \hat{{\textbf{c}}}}{{\textbf{1}}^\intercal \hat{{\textbf{c}}}} -1. \end{aligned}$$

The first term on the right hand side of Eq. (11) can be slightly rewritten, as

$$\begin{aligned} \frac{{\textbf{r}}^\intercal \hat{{\textbf{c}}}}{{\textbf{1}}^\intercal \hat{{\textbf{c}}}} = \sum _{i=1}^n \frac{c_i}{c_1\!+\!\ldots \!+\!c_n} \cdot r_i. \end{aligned}$$

This representation allows us to interpret this term as a weighted mean of the players’ productivities; the weights correspond to the players’ contributions. In particular, this observation implies that $E(\hat{{\textbf{c}}})$ is always in between $\min _i r_i - 1$ and $\max _i r_i - 1$. Whether or not the upper bound (or equivalently, the lower bound) can be realised depends on which players contribute in equilibrium. For example, the upper bound can be realised if and only if there is an equilibrium in which only those players *i* with $r_i = \max _j r_j$ make a contribution. That is only possible if there are multiple players with maximum productivity.

#### Example

It is instructive to illustrate these concepts with a two-player game (which we continue to use throughout this article). Consider the game $\Gamma _{\!\textrm{B}}\big ((1.5, 1.1)^\intercal , 0.9\big )$. That is, there are $n\!=\!2$ players with productivities $r_1 \!=\! 1.5$ and $r_2 \!=\! 1.1$ (as in Fig. 1), and the discount factor is $\delta \!=\! 0.9$. In this example, the value of the matrix *D* is

Suppose player 1 makes a constant contribution $\hat{c}_1\!=\!7$ in every round, whereas player 2 makes the constant contribution $\hat{c}_2\!=\!5$. It follows that the total size of the public good is ${\textbf{r}}^\intercal \hat{{\textbf{c}}} \!=\! 1.5\cdot 7 +1.1\cdot 5\!=\! 16$. Thus, each player’s share of the public good is 8, and their payoffs according to Eq. (3) are $\hat{\pi }_1\!=\!8\!-\!7\!=\!1$ and $\hat{\pi }_2\!=\!8\!-\!5\!=\!3$. Because both payoffs are non-negative, the respective constant contribution sequences are individually rational. Hence, by the folk theorem, they are sustainable in the repeated game for sufficiently large $\delta$. For this case of $n\!=\!2$, Eq. (6) in Corollary 3 takes the form of the following system of inequalities:

12 $$\begin{aligned} \hat{c}_1&\le \delta \frac{r_2}{2-r_1} \hat{c}_2 \end{aligned}$$

13 $$\begin{aligned} \hat{c}_2&\le \delta \frac{r_1}{2-r_2} \hat{c}_1 \end{aligned}$$

With these, we can verify that the given discount factor $\delta \!=\!0.9$ is indeed sufficiently large. According to Eq. (8), the resulting welfare is $W \!=\! 3 \!+\! 1\! =\! 4$, and according to Eq. (10), resource efficiency is $E \!=\! 4 / 12 \!\approx \! 0.333$. If contributions were ten times larger, welfare would increase to 40 but the resource efficiency would remain the same.

Instead, suppose now that the two players contribute equal constant amounts, say $\hat{c}_1 \!=\! \hat{c}_2 \!=\! 6$. By the inequalities (12–13), this contribution vector is also sustainable. It yields a payoff of 1.8 for each player. Thus, the welfare is $W \!=\! 3.6$, whereas resource efficiency is $E \!=\! 0.3$. We conclude that equal contributions are less resource efficient, compared to the previous example with unequal contributions. This is intuitive because in the previous example, the more productive player 1 contributed a larger share.

In light of these observations, it is natural to ask what the optimal ratio of the two player’s contributions is, if we aim to maximise resource efficiency in equilibrium. Again from the inequalities (12–13), we see that when $r_1\!>\!r_2$ (as in our example), this ratio is given by

$$\begin{aligned} \frac{\hat{c}_1}{ \hat{c}_2} = \delta \frac{r_2}{2 - r_1}. \end{aligned}$$

If for example $\hat{c}_1 \!=\! 10$, then $\hat{c}_2 \!=\! 500/99 \approx 5.051$, which yields payoffs of ${\hat{\pi }}_1 \!\approx \! 0.278$ and ${\hat{\pi }}_2 \!\approx \! 5.227$. The resulting welfare is $W \!\approx \! 5.505$ and resource efficiency is $E^\textrm{c}_{\sup } \!\approx \! 0.366$. Note that this maximum resource efficiency depends on the discount factor $\delta$. If $\delta$ were larger, even higher efficiencies would be possible. In contrast, if $\delta$ were lower, either $E^\textrm{c}_{\sup }$ would be lower, or the game might not allow for non-defection at all.

In the above example, we only considered the simple case of constant contributions. We now address the question of whether we can achieve higher resource efficiency when contribution sequences are allowed to be time-dependent.

### Efficiency with Time-Dependent Contributions

If we only consider the binary distinction between whether or not a game allows for non-defection, then constant and time-dependent contributions are equally effective. Specifically, if a game has any non-defective equilibrium at all, then it also has a non-defective equilibrium with a constant contribution sequence [Corollary 6 of 23]. However, below we show that within the space of non-defective equilibria, time-dependent contributions can indeed enable outcomes that are not sustainable otherwise. To this end, we first introduce some notation.

#### Notation 2

For two vectors ${\textbf{v}}$ and ${\textbf{w}}$, we write ${\textbf{v}} \!\le _1\! \textbf{w}$ if $v_i \!\le \! w_i$ for all *i* and $v_i \!=\! w_i$ for at most one *i*. In other words, ${\textbf{v}} \!\le _1\! {\textbf{w}}$ corresponds to ${\textbf{v}} \!\le \! {\textbf{w}}$ with equality in at most one component.

Using this notation, we can characterise which total contribution vectors are sustainable with time-dependent contribution sequences.

#### Theorem 5

Let $\Gamma _{\!\textrm{B}}({\textbf{r}}, \delta )$ allow for non-defection. Then the total contribution vector $\hat{{\textbf{c}}}$ is sustainable in game $\Gamma _{\!\textrm{B}}({\textbf{r}}, \delta )$ if and only if either $\hat{{\textbf{c}}} \!=\! {\textbf{0}}$ or

14 $$\begin{aligned} \hat{{\textbf{c}}} \le _1D \hat{{\textbf{c}}}. \end{aligned}$$

Furthermore, all sustainable total contribution vectors $\hat{{\textbf{c}}}$ are sustainable with a continuation contribution sequence $(\bar{{\textbf{c}}} (t))_t$ that satisfies $\hat{{\textbf{c}}} \le \bar{{\textbf{c}}} (t)$ for all *t*.

The proof of Theorem 5, and of all subsequent results, is provided in the Appendix.

Theorem 5 characterises the set of total contribution vectors that are sustainable in a given game $\Gamma _{\!\textrm{B}}({\textbf{r}}, \delta )$. The theorem is analogous to Corollary 3, which allowed for constant contribution sequences only. Interestingly, however, condition (6) in Corollary 3 depends on the discount rate $\delta$. In contrast, condition (14) is independent of $\delta$; the only requirement of the theorem is that $\delta$ be sufficiently large to allow for non-defection in the first place. That is, suppose the discount factor $\delta$ is large enough for the game to allow for *some* non-defective total contribution vector $\hat{{\textbf{c}}}$ to be sustainable. Then it automatically allows for *all* vectors that satisfy $\hat{{\textbf{c}}} \le _1D \hat{{\textbf{c}}}$.

The last part of the theorem states that the relevant contribution sequences can be chosen such that future contributions are always at least as large as the past contributions. This statement is included as a technical result that will be useful later on (in the proof of Proposition 14).

As a special case we obtain the following result on the sustainability of equal contributions across all players.

#### Corollary 6

Let $\Gamma _{\!\textrm{B}}({\textbf{r}}, \delta )$ allow for non-defection. Then for any $\lambda \ge 0$, the total contribution vector $\hat{{\textbf{c}}} = \lambda {\textbf{1}}$ is sustainable.

**Fig. 2:**
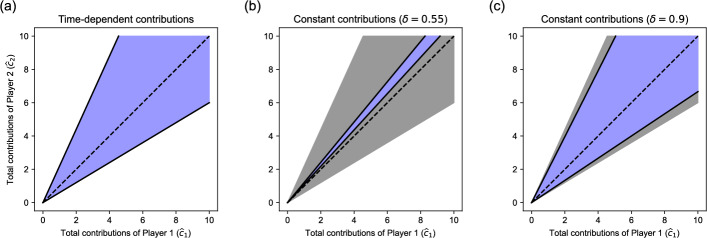
The region of sustainable total contribution vectors in a two-player game with $r_1 \!=\! 1.5$ and $r_2 \!=\! 1.1$. **a** The blue area shows the total contribution vectors that are sustainable with time-dependent contributions as long as $\delta$ is sufficiently large to allow any kind of non-defection, which in this case means $\delta \ge 0.522$. The dashed line represents equal contributions. **b** The blue area shows the total contribution vectors that are sustainable with constant contributions in the game $\Gamma _{\!\textrm{B}}({\textbf{r}}, 0.55)$. The grey area shows the total contribution vectors sustainable with time-dependent contributions, for comparison. **c** Like **b**, but for the game $\Gamma _{\!\textrm{B}}({\textbf{r}}, 0.9)$. The higher value of $\delta$ allows for a larger set of total contribution vectors to be sustainable with constant contributions

Importantly, for this statement to be true, time-dependent contributions are essential. If instead players are restricted to make constant contributions along the equilibrium path, there exist games $\Gamma _{\!\textrm{B}}({\textbf{r}}, \delta )$ where no equal contribution vector $\lambda {\textbf{1}}$ is sustainable [20]. Figure 2 illustrates these results. For our 2-player game with $r_1 \!=\! 1.5$ and $r_2 \!=\! 1.1$, it shows the region of total contribution vectors that are sustainable with time-dependent contribution sequences (Fig. 2a), which is independent of $\delta$, and the region of total contribution vectors that are sustainable with constant contribution sequences for a fixed value of $\delta$ (for $\delta \!=\!0.55$ in Fig. 2b and $\delta \!=\!0.9$ in Fig. 2c). Figure 2a was obtained from Eq. (14), which is independent of $\delta$. Figure 2b–c was obtained from the analogous condition for constant contribution sequences, Eq. (6), which depends on $\delta$. The figure shows that for a low discount factor like $\delta \!=\!0.55$, equal total contributions are feasible with a time-dependent contribution sequence , as predicted by Corollary 6, but not with a constant contribution sequence .

The contribution vectors $\hat{{\textbf{c}}}$ that are individually rational in the one-round game (in the sense of the folk theorem) are those where $\hat{{\textbf{c}}} \!\le \! D \hat{{\textbf{c}}}$. Theorem 5 shows that almost all of those (all except for a set with measure zero) are sustainable once the discount factor $\delta$ is sufficiently large to allow for *any* non-defective equilibrium. This requirement is considerably weaker than the one typically used in the folk theorem literature, where $\delta$ is thought to approach 1. In the case of two players, the above result holds for strictly all individually rational contribution vectors, as stated by Corollary 7 below.

#### Corollary 7

Let $n \!=\! 2$ and let $\Gamma _{\!\textrm{B}}({\textbf{r}}, \delta )$ allow for non-defection. Then the total contribution vector $\hat{{\textbf{c}}}$ is sustainable exactly if $\hat{{\textbf{c}}} \le D \hat{{\textbf{c}}}$.

Corollary 7 follows in two steps. First, we note that equality, $\hat{{\textbf{c}}} \!=\! D \hat{{\textbf{c}}}$, is impossible unless $\hat{{\textbf{c}}} = {\textbf{0}}$. To see why, first observe that $\hat{{\textbf{c}}} \!=\! D \hat{{\textbf{c}}}$ is equivalent to $n \hat{c}_i \!=\! \sum _j r_j \hat{c}_j$ for all players *i*. Summing up over all *i* and dividing by the group size *n* gives $\sum _j \hat{c}_j \! =\! \sum _j r_j \hat{c}_j$. Unless $\hat{{\textbf{c}}} = {\textbf{0}}$, this is a contradiction, because all $r_j$ are larger than one. The second step is straightforward: given that $\hat{{\textbf{c}}} \!\not =\! D \hat{{\textbf{c}}}$, the statements $\hat{{\textbf{c}}} \!\le \! D \hat{{\textbf{c}}}$ and $\hat{{\textbf{c}}} \!\le _1\! D \hat{{\textbf{c}}}$ are equivalent for $n\!=\!2$.

With Theorem 5, we have established that time-dependent contribution sequences can achieve total contribution vectors that are not sustainable with constant contributions. Because payoffs, resource efficiency, and welfare are all functions of the total contribution vector , we can use Theorem 5 to analyse the possible outcomes in terms of those quantities. In particular, the following result suggests that time-dependent contributions can indeed allow for a higher resource efficiency.

#### Theorem 8

Let $\Gamma _{\!\textrm{B}}({\textbf{r}}, \delta )$ allow for non-defection, let $r_{\max } \!=\! \max _i r_i$, and let *m* be the number of players with maximum productivity $r_{\max }$. There exists a sustainable time-dependent contribution sequence that is more resource-efficient than all sustainable constant contribution sequences if and only if

15 $$\begin{aligned} \big (1+\delta (m\!-\!1)\big ) \cdot r_{\max } < n. \end{aligned}$$

In particular, such a sequence exists if there is only a single player with productivity $r_{\max }$.

To understand the statement of Theorem 8, consider the case that there is a single player with maximum productivity (i.e., $m \!=\! 1$). In that case, (15) certainly holds, so the statement of the theorem is simply that resource efficiency cannot be optimised with constant contribution sequences.

Let us also provide some intuition for why the theorem holds, again by considering the case $m \!=\! 1$. Without loss of generality, let the unique most productive player be player 1. Let the group play the most resource-efficient sustainable constant contribution sequence (the maximum can indeed be attained). As shown in [23], this means that a player’s contribution is larger the more productive that player is; in particular, player 1’s contribution is the largest and hence positive. But in principle, player 1 could deviate and contribute nothing instead. That way, player 1 would get a positive payoff from the other players’ contributions in the first round, and a non-negative payoff thereafter. That is, player 1 would obtain a positive payoff overall. However because of our assumption that the contribution sequence is sustainable, player 1 does not benefit from such a deviation. It follows that player 1’s payoff from the constant contribution sequence is as least as good, hence positive. This means that the other players could decide to extract parts of this positive payoff from player 1. For example, they might require that in an initial extra round, player 1 make a solitary contribution. As long as the demanded contribution does not exceed player 1’s positive benefit from all subsequent contributions, player 1 would be compelled to go along.

Since this player is most productive, and now accounts for a greater share of the contributions, this new scheme increases resource efficiency.

**Fig. 3:**
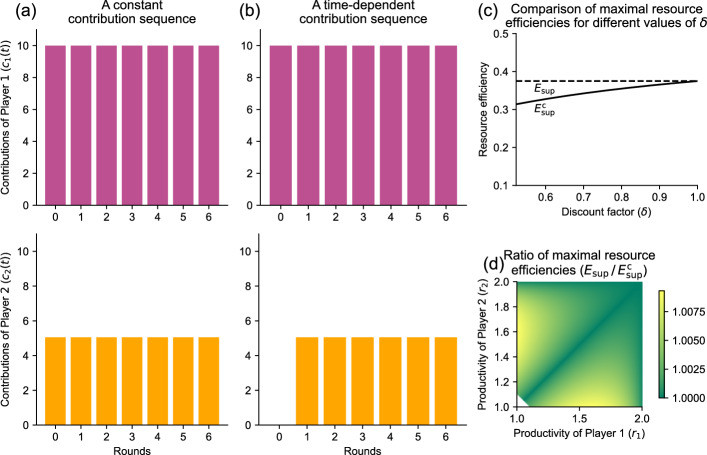
Time-dependent contributions allow for greater resource efficiency than constant contributions. Here, we illustrate this result with the game $\Gamma _{\!\textrm{B}}\big ((1.5, 1.1)^\intercal , 0.9\big )$. **a** When players are required to make constant contributions each round, maximum resource efficiency is attained for ${\hat{c}}_1 \!=\! 10$ and ${\hat{c}}_2 \!=\! 500/99 \!\approx \! 5.051$ (or any positive multiple thereof). The attained efficiency is $E^\textrm{c}_{\sup } \approx 0.366$. **b** Players can achieve a better resource efficiency of $E_{\sup } = 0.375$ by switching to a time-dependent contribution sequence. In this example, player 1’s contributions are still constant, while player 2’s contributions are constant after the first round. **c** For the same productivities as in (**a**) and (**b**), we show the optimal resource efficiency with constant contributions ($E^\textrm{c}_{\sup }$, solid line) and with any contributions ($E_{\sup }$, dashed line). (d) Here we depict the ratio $E_{\sup } / E^\textrm{c}_{\sup }$ for different productivities $r_1$ and $r_2$, for a discount factor of $\delta \! =\! 0.9$. The white area in the bottom left corner is where cooperation is not possible for this value of $\delta$

#### Example

Again, it is instructive to illustrate these considerations with our two-player example, game $\Gamma _{\!\textrm{B}}\big ((1.5, 1.1)^\intercal , 0.9\big )$. For constant contribution sequences, we have shown that the maximum resource efficiency is $E^\textrm{c}_{\sup } \approx 0.366$. This maximum is attained, for example, when the two players make contributions of ${\hat{c}}_1 \!=\! 10$ and ${\hat{c}}_2 \!=\! 500/99 \!\approx \! 5.051$ each round (or any positive multiple thereof), see Fig. 3a. By Theorem 8, time-dependent contribution sequences can achieve a superior resource efficiency, because there is a single player with maximum productivity. Figure 3b shows an example of such a time-dependent contribution sequence . Here, in round zero, player 1 contributes $c_1(0) \!=\! 10$ whereas player 2 contributes nothing. In all subsequent rounds, players use the numbers of the earlier constant contribution sequence, $c_1(t) \!=\! 10$ and $c_2(t) \!=\! 500/99 \!\approx \! 5.051$ for $t\!\ge \!1$. By Theorem 1, this contribution sequence is sustainable. Moreover, it achieves the best possible resource efficiency $E_{\sup } \!=\! 0.375$. This value of $E_{\sup }$ does not depend on the discount factor $\delta$, provided $\delta$ is sufficiently large to allow for non-defection in the first place (Fig. 3c). For the given ${\textbf{r}}$, one can compute this minimum discount factor to be $\delta _{\min } \approx 0.522$ ([23] In we show that $\delta _{\min }$ can be computed as the inverse of the largest eigen value of matrix *D*, as defined by Eq. (4)).

Interestingly, Theorem 8 also shows that in case of equal productivities for all players, there is no advantage from time-dependent contributions. To see why, observe that for $m\!=\!n$ and ${\textbf{r}} = (r, r, \dots , r)^\intercal$, condition (15) becomes

16 $$\begin{aligned} \big (1+\delta (n\!-\!1)\big ) \cdot r < n. \end{aligned}$$

However, by Theorem 2 in the SI Appendix of [23], $\Gamma _{\!\textrm{B}}(r, \delta )$ only allows for non-defection if

17 $$\begin{aligned} \delta \ge \frac{n-r}{r(n-1)}, \end{aligned}$$

Because condition (17) is the negation of (16), these two conditions are incompatible.

## Model Variation I: Endowment Constraints

So far, we considered the base model in which players were free to make arbitrary contributions each round. While this model has been convenient to work with, it has at least two disadvantages. First, it requires players to have access to arbitrary amounts of resources, which seems unrealistic. Second, it renders any attempt to optimise the players’ welfare meaningless, since players can arbitrarily scale up their contributions (and hence their welfare). In the following, we aim to show how the base model’s results extend to two more realistic model variants.

**Fig. 4:**
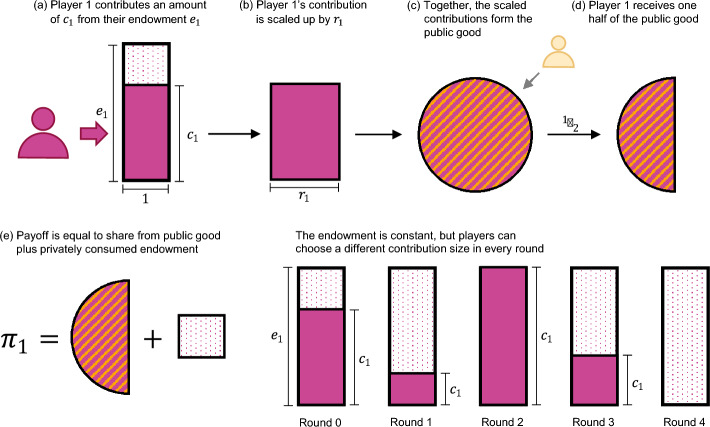
The endowment model. Here, the players’ contributions each round are constrained by their fixed endowment $e_i$. **a** In each round, players decide how much of their endowment they wish to contribute to the public good. The remaining amount $e_i \!-\! c_i$ is consumed privately. **b**–**d** The following steps are identical to the base model. In general, player 1 receives the *n*th share of the public good; in this example, $n=2$. **e** Players derive a payoff from their share of the public good, and from their private consumption of the remaining endowment

First, we consider a model variation called the endowment model. Here, there is a constant upper limit on player *i*’s contribution $c_i(t)$ in any given round. We refer to this upper limit as the player’s endowment, $e_i$, and we use the notation ${\textbf{e}} \!=\! (e_1,\ldots ,e_n)$ to refer to the vector of all endowments. Endowments are positive and, without loss of generality, normalised such that $\sum _i e_i \!=\! 1$. In each round *t*, each player *i* chooses which part of their endowment to contribute. That is, they choose some $c_i(t) \in [0, e_i]$. Contributions have the same effect as in the base model. Overall, the players’ payoffs consist of their share of the public good, and of their remaining endowment (see Fig. 4),

$$\begin{aligned} \pi _i(t) = \frac{1}{n} {\textbf{r}}^\intercal {\textbf{c}}(t)+ \big (e_i \!-\! c_i(t)\big ). \end{aligned}$$

Note that in the case of zero contributions, we now have payoffs of $\hat{\varvec{\uppi }} \!=\! {\textbf{e}}$, as opposed to the base model, where zero contributions give $\hat{\varvec{\uppi }} \!=\! {\textbf{0}}$. However, the definition of welfare remains the same as before, $W = \sum _{i=1}^n {\hat{\pi }}_i$. We define resource efficiency for this model as

$$\begin{aligned} E = \frac{\sum _{i=1}^n ({\hat{\pi }}_i - e_i)}{\sum _{i=1}^n c_i}, \end{aligned}$$

that is, sum of obtained payoffs minus full defection payoffs, divided by the sum of all contributions. Eq. (11) remains valid.

### Notation 3

In the endowment model, a game is specified by the players’ productivities ${\textbf{r}}$, their endowments ${\textbf{e}}$, and the discount factor $\delta$. We denote the respective game as $\Gamma _{\!\textrm{E}}({\textbf{r}}, {\textbf{e}}, \delta )$.

We can now translate the results of the base model to the endowment model. Of Corollary 6, we obtain a weaker version:

### Corollary 9

Let $\Gamma _{\!\textrm{E}}({\textbf{r}}, {\textbf{e}}, \delta )$ allow for non-defection. Then there exists a $\lambda \!>\! 0$ such that equal contributions of $\hat{{\textbf{c}}} \!=\! \lambda {\textbf{1}}$ are feasible and sustainable.

In other words, if any kind of non-defection is possible, then equal total contributions are also possible. However, now these contributions need to be sufficiently small so that in each round, the players do not exceed their endowment limits. Similar to the base model, the statement of Corollary 9 does no longer hold when we require contributions to be constant [20]. That is, there are cases when equal total contributions are possible, but only with time-dependent contribution sequences .

Trivially from Corollary 2, whether or not non-defection is possible in $\Gamma _{\!\textrm{E}}({\textbf{r}}, {\textbf{e}}, \delta )$ does not depend on ${\textbf{e}}$; rather, it is possible if and only if it is possible in $\Gamma _{\!\textrm{B}}({\textbf{r}}, \delta )$. If that is the case, we may simply say that the pair $({\textbf{r}}, \delta )$ allows for non-defection, without referring to a particular one of the models.

Since resource efficiency is invariant under linear scalings of the contributions, all sustainable resource efficiencies can be achieved with arbitrarily small contributions. The set of sustainable resource efficiencies is thus unaffected by endowment constraints. This insight trivially allows us to extend the results about resource efficiency from the base model to the endowment model, as formally stated in the following proposition.

### Proposition 10

Take any game ${\textbf{r}}$, ${\textbf{e}}$, $\delta$.

1. The set of sustainable resource efficiencies in $\Gamma _{\!\textrm{E}}({\textbf{r}}, {\textbf{e}}, \delta )$ is the same as in $\Gamma _{\!\textrm{B}}({\textbf{r}}, \delta )$.
2. The set of resource efficiencies sustainable with constant contributions in $\Gamma _{\!\textrm{E}}({\textbf{r}}, {\textbf{e}}, \delta )$ is the same as in $\Gamma _{\!\textrm{B}}({\textbf{r}}, \delta )$.

It follows that Theorem 8 equally applies to the endowment model. In particular, for any game $\Gamma _{\!\textrm{E}}({\textbf{r}}, {\textbf{e}}, \delta )$, time-dependent contributions can enable higher resource efficiencies than constant contributions.

In addition to resource efficiency, in the endowment model it is also meaningful to study the player’s welfare. However, with respect to welfare, the following (existing) result shows that time-dependent contributions provide no advantage.

### Proposition 11

( [23], Supplementary Information Lemma 2) In any game $\Gamma _{\!\textrm{E}}({\textbf{r}}, {\textbf{e}}, \delta )$, sustainable welfare attains a maximum, and that maximum is attained with constant contributions.

### Example

To illustrate how the endowment model relates to the base model, we consider a game with the same parameters as in the previous example, that is, with ${\textbf{r}}\!=\!(1.5, 1.1)^\intercal$ and $\delta \!=\!0.9$. As the endowment distribution, we choose $\textbf{e}\!=\!(0.2, 0.8)^\intercal$. So we have the game $\Gamma _{\!\textrm{E}}((1.5, 1.1)^\intercal , (0.2, 0.8)^\intercal , 0.9)$.

In the previous example, we saw that the highest attainable resource efficiency in $\Gamma _{\!\textrm{B}}((1.5, 1.1)^\intercal , 0.9)$ is $E_{\sup }\!=\!0.375$ and that it is only attainable with a time-dependent contribution sequence. By Proposition 10, these statements carry over identically to the endowment model independently of our choice of ${\textbf{e}}$. The sequence we constructed earlier to obtain resource efficiency 0.375 was given by $c_1(0)\!=\!c_1(1)\!=\!c_1(2)\!=\!\dots \!=\!10$ and $c_2(0)\!=\!0$ and $c_2(1)\!=\!c_2(1)\!=\!\dots \!=\!500/99$. Of course, contributions of that size would by far exceed both players’ endowments. But we can scale the sequence down by a factor of 50 to get $c_1(0)\!=\!c_1(1)\!=\!c_1(2)\!=\!\dots \!=\!0.2$ and $c_2(0)\!=\!0$ and $c_2(1)\!=\!c_2(1)\!=\!\dots \!=\!10/99\!\approx \!0.101$, which is feasible with respect to the endowments, and sustainable in equilibrium. This sequence has the same resource efficiency of 0.375.

However, while player 1 contributes their entire endowment of 0.2 in every round, the largest part of player 2’s endowment is unproductive in this contribution sequence. This is reflected in the comparatively low welfare. In round 0, payoffs are $\pi _1(0)\!=\!0.15 + (0.2-0.2)\!=\!0.15$ and $\pi _2(0)\!=\!0.15\!+\!(0.8\!-\!0) =\!0.95$. In the subsequent rounds, payoffs are $\pi _1(t) \!\approx \! 0.205 + (0.2-0.2)\!\approx \!0.205$ and $\pi _2(t)\!\approx \!0.205 + (0.8-0.101)\!\approx \!0.904$. This gives an overall welfare of $W\!\approx \!1.108$ (remember that here, the welfare of full defection is $W\!=\!1$). Compare that to constant contributions of $\hat{c}_1\!=\!0.2$ and $\hat{c}_2\!=\!0.3$, which are sustainable and achieve an optimal welfare of $W\!=\!1.13$. Proposition 11 states that the welfare optimum can always be attained with constant contributions.

There is an intuitive general reason why time-dependent contributions have no positive welfare effects: In the endowment model, the more a contribution sequence varies, the more resources are non-productively withheld in some of the rounds. In particular, only constant contributions can achieve full cooperation, which by definition means $c_i(t)\!=\!e_i$ for all *t* and *i*. (With the right endowment distribution, a maximal welfare of $W\!=\!E^\textrm{c}_{\sup }\!+\!1$ can be realised that way; in the above example that is $W\!\approx \!1.366$.) This observation suggests that the endowment model is inherently geared towards constant contributions. Instead, we would like to consider a setup in which overall contributions are constrained, yet players may freely choose how to allocate their contributions over time. To do this, we introduce another model variant.

## Model Variation II: A Model with Savings

The savings model builds on the earlier endowment model. Again, each player *i* obtains a fixed endowment $e_i$ every round. However, now players have three options for how to spend their endowment, rather than two. They can contribute to the public good, consume parts or all of the endowment privately, or they can make a deposit into a savings account. Savings pay interest at the rate of $(\delta ^{-1} \!-\!1)$ per round – which exactly corresponds to the time value of money at the discount factor $\delta$. These savings can then be spent in future rounds, either to contribute to the public good or for private consumption.

More specifically, each round proceeds as follows. In the beginning of each round *t*, players receive an endowment $e_i$. In addition, they have access to an amount of $s_i(t)$ on their savings account (in the very first round, savings are set to zero). Players then decide which amount $p_i(t)$ to consume privately, which amount $c_i(t)$ to contribute to the public good, and which amount $d_i(t)$ to deposit into the savings account. These variables need to satisfy the budget constraint $e_i\!+\!s_i(t) = p_i(t)\!+\!c_i(t)\!+\!d_i(t)$. Contributions to the public good and private consumption directly enter the player’s payoff function,

$$\begin{aligned} \pi _i(t) = \frac{1}{n} {\textbf{r}}^\intercal {\textbf{c}}(t) +p_i(t). \end{aligned}$$

**Fig. 5:**
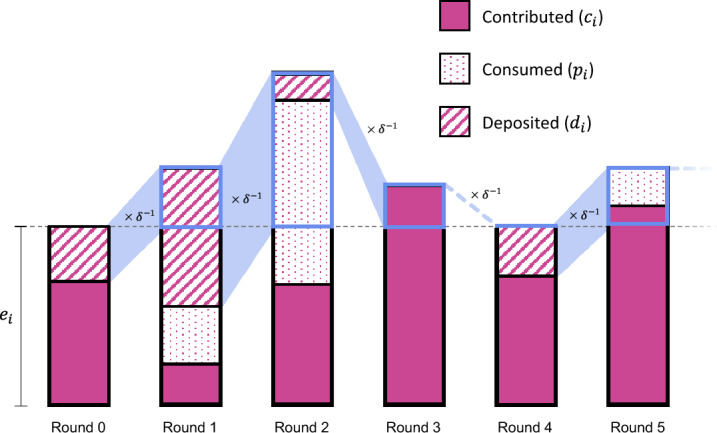
The savings model. Here, we depict the first six rounds of a player’s gameplay. In each round, the player not only has their endowment $e_i$ available, but also whatever resources they deposited in the previous round, plus interest at the rate $\delta ^{-1}\!-\!1$. They decide which part of that they want to contribute, which part to consume, and which part to deposit to the savings account

Deposits, on the other hand, determine a player’s savings in the beginning of the next round, $s_i(t\!+\!1) \!=\! \delta ^{-1} d_i(t)$. At time $t\!+\!1$, the process is repeated; individuals again have to decide how much to consume, to contribute, and to save, see Fig. 5. Total payoffs (across all rounds), welfare, and resource efficiency are then defined as in the previous two models.

### Notation 4

The savings model uses the same parameters as the endowment model: productivities ${\textbf{r}}$, endowments ${\textbf{e}}$, and the discount factor $\delta$. We denote the game as $\Gamma _{\!\textrm{S}}({\textbf{r}}, {\textbf{e}}, \delta )$.

We make the following observations about the savings model: First, in our implementation of this model, savings are payoff-neutral: The interest earned over one round is exactly offset by a player’s discounting of future rewards. Second, without the opportunity for saving, this model recovers the endowment model as discussed in the previous section and studied in [20]. Third, the savings model is equivalent to saying that endowments only apply as a constraint to the cumulative contributions. That is, each player *i* is required to play such that $\sum _{\tau =0}^t \delta ^{\tau } c_i(t) \!\le \! \sum _{\tau =0}^t \delta ^{\tau } e_i$ for all *t*, but without the stronger requirement that $c_i(t) \!\le \! e_i$ for all *t*. Finally, since resource efficiency is equal to surplus welfare divided by total contributions, maximising resource efficiency in the base model is equivalent to maximising welfare in the savings model when the endowments are also an optimisation variable.

To state our main results for this section, we first define the notion of a welfare supremum with and without savings. For given parameters ${\textbf{r}}, {\textbf{e}}, \delta$, the welfare supremum with savings $W_{\sup }^{\textrm{s}}({\textbf{e}})$ is the supremum of welfare over all equilibria of the game $\Gamma _{\!\textrm{S}}({\textbf{r}}, {\textbf{e}}, \delta )$. It quantifies the maximum value that the group can derive from cooperation. Similarly, we define the welfare supremum without savings, $W_{\sup }({\textbf{e}})$, as the supremum of welfare over all equilibria that satisfy $d_i(t) \!=\! 0$ for all *i* and *t*. It quantifies the maximum value that the group can derive without ever saving any amount. Equivalently, it corresponds to the welfare supremum of the endowment model, $\Gamma _{\!\textrm{E}}({\textbf{r}}, {\textbf{e}}, \delta )$. We interpret the difference $W_{\sup }^{\textrm{s}}({\textbf{e}}) - W_{\sup }({\textbf{e}})$ as the (positive or zero) advantage that savings can provide.

### Theorem 12

Let $({\textbf{r}}, \delta )$ allow for non-defection. Take any endowment distribution ${\textbf{e}}$. Then savings provide no advantage (i.e. $W_{\sup }^{\textrm{s}}({\textbf{e}}) \!=\! W_{\sup }({\textbf{e}})$), if and only if

18 $$\begin{aligned} {\textbf{e}} \le \delta D {\textbf{e}}. \end{aligned}$$

Without savings, $\hat{{\textbf{c}}} = {\textbf{e}}$ requires constant contributions. So by Corollary 3, Eq. (18) is equivalent to $\hat{{\textbf{c}}} = {\textbf{e}}$ being sustainable without savings. Therefore, Theorem 12 states that exactly one of the following is the case: Either full contributions are sustainable without savings, or savings provide an advantage for welfare. In particular, for any ${\textbf{r}}$ and ${\textbf{e}}$, when $\delta$ is sufficiently low, savings provide an advantage. Alternatively, for any ${\textbf{r}}$ and $\delta$, when ${\textbf{e}}$ is sufficiently unequal, savings provide an advantage ( [20], Supplementary Information Proposition 3).

Savings also provide an advantage from the perspective of a social planner who chooses an endowment distribution with the aim of maximising welfare. To assess this, we consider the suprema of $W_{\sup }^{\textrm{s}}({\textbf{e}})$ and $W_{\sup }({\textbf{e}})$ over all possible endowment distributions ${\textbf{e}}$. From Theorem 8, we can derive the following result:

### Theorem 13

Let $({\textbf{r}}, \delta )$ allow for non-defection. Let $r_{\max } = \max _i r_i$, and let *m* be the number of players with productivity $r_{\max }$. Then $\sup _{{\textbf{e}}} W_{\sup }^{\textrm{s}}({\textbf{e}}) > \sup _{{\textbf{e}}} W_{\sup }({\textbf{e}})$ if and only if

19 $$\begin{aligned} \big (1+\delta (m\!-\!1)\big ) \cdot r_{\max } < n. \end{aligned}$$

To understand the theorem intuitively, consider again the case that there is only a single player with maximum productivity $r_{\max }$, i.e. that $m\!=\!1$. Then Theorem 13 states that strictly better welfare is possible when players are permitted to save part or all of their endowment for later rounds, compared to when they are not. This is because with saving, they can play a more resource-efficient time-dependent contribution sequence and still productively use all of their endowment, whereas without, they are restricted to make constant contributions in order to maximise welfare. Conversely, as another special case of Theorem 13, we conclude that savings never provide a welfare advantage if all players have the same productivity.

### Example

We revisit the same example as before, now in the savings model: $\Gamma _{\!\textrm{S}}((1.5, 1.1)^\intercal , (0.2, 0.8)^\intercal , 0.9)$. In the endowment model, we had to scale the optimally resource-efficient contribution sequence to $c_1(0)\!=\!0.2$ so that players do not exceed their endowment limits. With savings, it is enough that at no point in time their cumulative contributions exceed the endowment limit. A simple example of a superior contribution sequence is as follows. In round 0, player 1 contributes and consumes nothing ($c_1(0)\!=\!p_1(0)\!=\!0$) and deposits everything ($d_1(0)\!=\!e_1\!=\!0.2$). In round 1, with the interest received, player 1 has savings of approximately 0.222 and again receives an endowment of 0.2, which makes for a total available amount of 0.422. Of this, player 1 contributes $c_1(1)\!\approx \!0.222$ and again deposits $d_1(1)\!=\!0.2$. In round 2, savings with interest again make up 0.222, and player 1 continues with contributing 0.222 and depositing 0.2 in every subsequent round. Player 2, on the other hand, from the beginning simply contributes $c_2(t)\!\approx \!0.333$ in every round and privately consumes the rest, $p_2(t)\!\approx \!0.467$, without depositing anything. The welfare of this contribution sequence is $W\!\approx \!1.164$, which is more than the optimal value without saving, $W\!=\!1.13$. Theorem 12 predicts that saving provides an advantage like this as long as full contributions are not sustainable, which is the case here.

The optimal welfare over all endowment distributions, $\sup _{{\textbf{e}}} W_{\sup }^{\textrm{s}}({\textbf{e}})$, requires the endowment distribution . This is exactly the endowment distribution at which the maximally resource efficient contribution sequence can be played in such a way that all endowments are eventually contributed: Player 1 contributes $c_1(t)\!=\!11/16$ in every round. Player 2 deposits everything in round 0 ($d_2(0)\!=e_2\!=\!5/16$). Thereafter, player 2 contributes $50/(16\cdot 9)$ and deposits 5/16 in every round. (The sequence of contributions is identical to that of the earlier example in Sect. 2, up to rescaling by a factor of 160/11, and thus also maximally resource efficient.) In this sequence, all resources are used productively and none are consumed privately, which means that the optimal resource efficiency also translates to optimal welfare. Indeed, the welfare is $W\!=\!\sup _{{\textbf{e}}} W_{\sup }^{\textrm{s}}({\textbf{e}}) = 1 + E_{\sup } = 1.375$. The fact that this is greater than the optimum without saving over all endowment distributions, which by Proposition 11 is $\sup _{{\textbf{e}}} W_{\sup }({\textbf{e}}) = 1 + E^{\mathrm c}_{\sup } \approx 1.366$, is predicted by Theorem 13.

## Discussion

The repeated public good game is one of the major models in (evolutionary) game theory to understand cooperation in groups. This literature describes how individuals can use conditionally cooperative strategies to sustain outcomes that are infeasible in one-shot encounters. Yet when describing the possible equilibrium outcomes, many previous studies implicitly restrict their analysis to the case that players make the same constant contribution each round [e.g. 20, 23]. Instead, here we study the effect of time-dependent contributions. Contrary to many other models of reciprocity, we allow players to select their actions from a continuum between full defection and full cooperation. We explore to which extent individuals can obtain better outcomes (e.g., a better resource efficiency or welfare) when they are able to vary their contributions along the equilibrium path.

From the outset, it is not clear whether time-dependent contributions provide any substantial advantage at all. After all, suppose players could achieve a superior outcome with contributions $\big ({\textbf{c}}(t)\big )_t$ that vary in time. Then players might achieve just the same outcome by instead making a constant contribution $\hat{{\textbf{c}}}$ each round, where $\hat{{\textbf{c}}}$ is the appropriate (time-discounted) average contribution per round, $\hat{{\textbf{c}}} = (1\!-\!\delta ) \sum _t \delta ^t c(t)$. With respect to their payoff implications, the two sequences $\big ({\textbf{c}}(t)\big )_t$ and $(\hat{{\textbf{c}}})_t$ are identical. After all, by Eq. (3), payoffs only depend on the players’ total contributions across all rounds. As a result, the two sequences generate the same resource efficiency and welfare. However, as we show in this article, the two sequences may differ in their sustainability. There are instances in which the time-dependent sequence $\big ({\textbf{c}}(t)\big )_t$ can be realised by a subgame perfect equilibrium, whereas the constant sequence $(\hat{{\textbf{c}}})_t$ cannot.

To make this point, we study three different models: a base model, a model with endowment constraints, and a model with savings. In the base model, players are allowed to make arbitrary contributions each round (the only requirement is that the sequence of contributions does not diverge). This setup imposes minimal constraints on the players’ behaviour, and it is convenient to work with mathematically. In contrast, the other two models are perhaps more realistic (and hence they have been studied more frequently). For example, the endowment model corresponds to the classical setup that is also frequently used in experiments [e.g. 6, 10, 21, 28]. Here, contributions are constrained by the endowments that the players receive each round. The savings model is similar, but in addition it allows players to (payoff-neutrally) transfer some of their endowments to future rounds. Interestingly, many of our results for these last two model variants are directly related to our findings in the base model. As an example, with Theorem 13, we characterise under which circumstances savings provide a welfare advantage in the savings model. The respective result is directly related to whether or not time-dependent contributions provide an advantage in the base model, Theorem 8. These similarities between those theorems highlight how several findings in the more abstract base model carry over to more applied settings.

Interestingly, the respective theorems also suggest that for our results, some asymmetry among players is crucial. Specifically, when players are identical with respect to their productivities, Theorem 8 shows that time-dependent contributions do not grant any advantage. Any resource efficiency that can be sustained with time-dependent contributions can already be sustained with constant contributions. But once players differ in their productivities, it becomes fairly easy for time-dependent contributions to be superior. In fact, such an advantage is guaranteed when the group contains a single player whose productivity exceeds everyone else’s.

Overall, our findings highlight the impact of variable contributions on resource efficiency and, more generally, on the sustainability of cooperation.

They suggest that by focussing solely on constant contributions, we may overlook important equilibria that can arise in dynamic settings.

## Data Availability

No datasets were generated or analysed during the current study.

## Appendix A Proofs

### A.1 Proof of Theorem 5

#### Proof of Theorem 5

Clearly $\hat{{\textbf{c}}} = {\textbf{0}}$ is sustainable, so it is sufficient to show the statement for $\hat{{\textbf{c}}} \not = {\textbf{0}}$.

First we will show that any sustainable $\hat{{\textbf{c}}} \not = {\textbf{0}}$ satisfies $\hat{{\textbf{c}}} \le _1D \hat{{\textbf{c}}}$. By Theorem 1, we have for any sustainable $\bar{\textbf{c}}(t)$ that

A1 $$\begin{aligned} \delta \bar{{\textbf{c}}} (1) \le \bar{{\textbf{c}}}(0) \le \delta D \bar{{\textbf{c}}}(1). \end{aligned}$$

Left-multiplying with the non-negative matrix *D* on both sides of the first inequality, we obtain

$$\begin{aligned} \delta D \bar{{\textbf{c}}} (1) \le D \bar{{\textbf{c}}}(0) \end{aligned}$$

with equality in the *i*th component exactly if $\delta c_j(1) = c_j(0)$ for all $j \not = i$.

Together with the second inequality of (A1), this gives

$$\begin{aligned} \bar{{\textbf{c}}}(0) \le D \bar{{\textbf{c}}}(0), \end{aligned}$$

or equivalently

A2 $$\begin{aligned} \hat{{\textbf{c}}} \le D \hat{{\textbf{c}}}, \end{aligned}$$

where equality in the *i*th component requires $\delta c_j(1) = c_j(0)$ for all $j \not = i$.

If (A2) has equality in at least two components, then $\delta c_j(1) = c_j(0)$ for all *j*, so $\delta {\textbf{c}}(1) = \textbf{c}(0)$. Analogously, equality in two components requires $\delta \textbf{c}(2) = {\textbf{c}}(1)$, etc., so the sequence $({\textbf{c}}(t))_t$ diverges and is not a valid contribution sequence . That is a contradiction, so we can have ${\hat{c}}_i = (D \hat{{\textbf{c}}})_i$ for at most one *i*.

Now we will show that if some $\hat{{\textbf{c}}} \not = {\textbf{0}}$ satisfies $\hat{{\textbf{c}}} \le _1D \hat{{\textbf{c}}}$, then $\hat{{\textbf{c}}}$ is sustainable with a continuation contribution sequence $(\bar{{\textbf{c}}} (t))_t$ that satisfies $\hat{{\textbf{c}}} \le \bar{{\textbf{c}}} (t)$ for all *t*. Assume first that the stronger condition ${\textbf{0}}< \hat{{\textbf{c}}} < D \hat{{\textbf{c}}}$ holds.

Take $\varepsilon > 0$ such that $1+\varepsilon < \delta ^{-1}$ and $(1+\varepsilon ) {\textbf{x}} \le D {\textbf{x}}$. Let ${\textbf{v}}$ be the Perron eigenvector of *D*, scaled so that ${\textbf{v}} \le {\textbf{x}}$. Let finally

$$\begin{aligned} T = \lceil \frac{\delta _{\textrm{min}} \max _i \frac{x_i}{v_i} - \min _i \frac{x_i}{v_i}}{\varepsilon ( 1 - \delta _{\textrm{min}} ) } \rceil \end{aligned}$$

or $T = 0$, whichever is larger.

Let ${\textbf{x}} = \hat{{\textbf{c}}}$ for a given $\hat{{\textbf{c}}}$ with ${\textbf{0}}< \hat{{\textbf{c}}} < D \hat{{\textbf{c}}}$. We define a continuation contribution sequence $(\bar{\textbf{c}}(t))_t$ by

$$\begin{aligned} \bar{{\textbf{c}}}(t) = ((1+\varepsilon ) \delta )^{-t} ( {\textbf{x}} + \varepsilon t {\textbf{v}} ) \end{aligned}$$

for all $0 \le t \le T$ and

$$\begin{aligned} \bar{{\textbf{c}}}(t) = (1+\varepsilon )^{-T} \delta ^{-(T+1)} \left( \min _i \frac{x_i}{v_i} + \varepsilon T \right) {\textbf{v}} \end{aligned}$$

for all $t > T$. If we show that it obeys (5) for all *t*, we know it is sustainable. Since $\bar{{\textbf{c}}}(0) = {\textbf{x}}$, that is enough to prove the first statement of the present theorem. We also see that $(\bar{{\textbf{c}}}(t))_t$ is non-decreasing. Therefore, $\hat{{\textbf{c}}} = \bar{{\textbf{c}}}(0) \le \bar{{\textbf{c}}}(t)$ for all *t*, which is the second statement of the theorem.

For $0 \le t < T$, the first inequality, $\delta \bar{{\textbf{c}}}(t+1) \le \bar{{\textbf{c}}}(t)$, follows from

$$\begin{aligned} {\textbf{v}} \le {\textbf{x}} \end{aligned}$$

as follows. First, we multiply with $\varepsilon$ and add $\varepsilon ^2 t {\textbf{v}}$, which is non-negative, on the right-hand side:

$$\begin{aligned} \varepsilon {\textbf{v}} \le \varepsilon {\textbf{x}} + \varepsilon ^2 t {\textbf{v}} \end{aligned}$$

We add ${\textbf{x}} + \varepsilon t {\textbf{v}}$ on both sides and factor out:

$$\begin{aligned} {\textbf{x}} + \varepsilon (t+1) {\textbf{v}} \le (1+\varepsilon )( {\textbf{x}} + \varepsilon t {\textbf{v}} ) \end{aligned}$$

We multiply with $\delta ((1+\varepsilon ) \delta )^{-(t+1)}$ on both sides:

$$\begin{aligned} \delta ((1+\varepsilon ) \delta )^{-(t+1)}( {\textbf{x}} + \varepsilon (t+1) {\textbf{v}} ) \le ((1+\varepsilon ) \delta )^{-t}( {\textbf{x}} + \varepsilon t \textbf{v}) \end{aligned}$$

This is equivalent to

$$\begin{aligned} \delta \bar{{\textbf{c}}}(t+1) \le \bar{{\textbf{c}}}(t) \end{aligned}$$

by the definition of $\bar{{\textbf{c}}}(t)$.

The second inequality, $\bar{{\textbf{c}}}(t) \le \delta D \bar{\textbf{c}}(t+1)$, follows from

$$\begin{aligned} {\textbf{v}} = \delta _{\textrm{min}} D {\textbf{v}}. \end{aligned}$$

We replace $\delta _{\textrm{min}}$ with $\delta$, which is larger or equal, on the right-hand side:

$$\begin{aligned} {\textbf{v}} \le \delta D {\textbf{v}} \end{aligned}$$

By construction, $1 - \varepsilon < \delta ^{-1}$, so we can write:

$$\begin{aligned} (1+\varepsilon ) {\textbf{v}} < D {\textbf{v}} \end{aligned}$$

Multiply by $\varepsilon t$ on the left-hand side and by $\varepsilon (t+1)$ on the right-hand side:

$$\begin{aligned} (1+\varepsilon ) \varepsilon t {\textbf{v}} < \varepsilon (t+1) D {\textbf{v}} \end{aligned}$$

Sum with the inequality $(1+\varepsilon ) {\textbf{x}} \le D {\textbf{x}}$, which also holds by construction of $\varepsilon$:

$$\begin{aligned} (1+\varepsilon ) ({\textbf{x}} + \varepsilon t {\textbf{v}}) < D ({\textbf{x}} + \varepsilon (t+1) {\textbf{v}}) \end{aligned}$$

Multiply by $\delta ((1+\varepsilon ) \delta )^{-(t+1)}$ on both sides:

$$\begin{aligned} ((1+\varepsilon ) \delta )^{-t} ( {\textbf{x}} + \varepsilon t {\textbf{v}} ) < \delta ((1+\varepsilon ) \delta )^{-(t+1)} D ( {\textbf{x}} + \varepsilon (t+1) {\textbf{v}} ) \end{aligned}$$

This is equivalent to

$$\begin{aligned} \bar{{\textbf{c}}}(t) \le \delta D \bar{{\textbf{c}}}(t+1). \end{aligned}$$

Next, we consider $t = T$: The first inequality follows from

$$\begin{aligned} {\textbf{x}} + \varepsilon T {\textbf{v}} \le (1+\varepsilon ) ( {\textbf{x}} + \varepsilon T {\textbf{v}} ). \end{aligned}$$

First, we replace ${\textbf{x}}$ by $\min _i \frac{x_i}{v_i} {\textbf{v}}$, which is at most as large in each component:

$$\begin{aligned} \min _i \frac{x_i}{v_i} {\textbf{v}} + \varepsilon T {\textbf{v}} \le (1+\varepsilon ) ( {\textbf{x}} + \varepsilon T {\textbf{v}} ) \end{aligned}$$

Then, we multiply by $\delta ((1+\varepsilon ) \delta )^{-(T+1)}$ on both sides:

$$\begin{aligned} \delta ((1+\varepsilon ) \delta )^{-(T+1)} \left( \min _i \frac{x_i}{v_i} + \varepsilon T \right) {\textbf{v}} \le ((1+\varepsilon ) \delta )^{-T} ( {\textbf{x}} + \varepsilon T {\textbf{v}} ) \end{aligned}$$

This is by definition equivalent to

$$\begin{aligned} \delta \bar{{\textbf{c}}}(T+1) \le \bar{{\textbf{c}}}(T). \end{aligned}$$

The second inequality follows from

$$\begin{aligned} \lceil \frac{\delta _{\textrm{min}} \max _i \frac{x_i}{v_i} - \min _i \frac{x_i}{v_i}}{\varepsilon ( 1 - \delta _{\textrm{min}} ) } \rceil \le T, \end{aligned}$$

which is by definition of *T*. We remove the ceiling function and multiply by $\varepsilon (1 - \delta _{\textrm{min}})$ on both sides:

$$\begin{aligned} \delta _{\textrm{min}} \max _i \frac{x_i}{v_i} - \min _i \frac{x_i}{v_i} \le (1 - \delta _{\textrm{min}}) \varepsilon T \end{aligned}$$

We move $\min _i \frac{x_i}{v_i}$ to the right-hand side, $\delta _{\textrm{min}} \varepsilon T$ to the left, and then multiply by $\delta _{\textrm{min}}^{-1} {\textbf{v}}$:

$$\begin{aligned} \max _i \frac{x_i}{v_i} {\textbf{v}} + \varepsilon T {\textbf{v}} \le \left( \min _i \frac{x_i}{v_i} + \varepsilon T \right) \delta _{\textrm{min}}^{-1} {\textbf{v}} \end{aligned}$$

Now, we can replace $\max _i \frac{x_i}{v_i} {\textbf{v}}$ by ${\textbf{x}}$, which is at most as large in each component, and $\delta _{\textrm{min}}^{-1} {\textbf{v}}$ by $D {\textbf{v}}$, which is equal:

$$\begin{aligned} {\textbf{x}} + \varepsilon T {\textbf{v}} \le \left( \min _i \frac{x_i}{v_i} + \varepsilon T \right) D {\textbf{v}} \end{aligned}$$

We multiply by $((1+\varepsilon ) \delta )^{-T}$ on both sides:

$$\begin{aligned} ((1+\varepsilon ) \delta )^{-T} ( {\textbf{x}} + \varepsilon T {\textbf{v}} ) \le \delta D \left( (1+\varepsilon )^{-T} \delta ^{-(T+1)} \left( \min _i \frac{x_i}{v_i} + \varepsilon T \right) {\textbf{v}} \right) \end{aligned}$$

This is by definition equivalent to

$$\begin{aligned} \bar{{\textbf{c}}}(t) \le \delta D \bar{{\textbf{c}}}(t+1). \end{aligned}$$

Finally, for $t > T$, we have $\bar{{\textbf{c}}}(t) = \bar{{\textbf{c}}}(t+1)$. The first inequality is trivially true, and the second inequality is true since $\bar{{\textbf{c}}}(t)$ is a multiple of ${\textbf{v}}$. So we have shown that $\hat{{\textbf{c}}}$ is sustainable if ${\textbf{0}}< \hat{{\textbf{c}}} < D \hat{{\textbf{c}}}$.

Now we will reduce the more general case of $\hat{{\textbf{c}}} \not = \textbf{0}$ and $\hat{{\textbf{c}}} \le _1D \hat{{\textbf{c}}}$ to this in two sequential steps.

First, let some $\hat{{\textbf{c}}} \not = {\textbf{0}}$ have ${\textbf{0}} < \hat{{\textbf{c}}} \le _1D \hat{{\textbf{c}}}$ with equality of the weak inequality in exactly one component *i*. Let ${\textbf{x}}(\varepsilon ) = \hat{{\textbf{c}}} - \varepsilon {\textbf{u}}_i$, where ${\textbf{u}}_i$ is the *i*th standard unit vector. Since ${\textbf{x}}(\varepsilon ) \rightarrow \hat{{\textbf{c}}}$ as $\varepsilon \rightarrow \infty$ and $\hat{c}_j < (D \hat{{\textbf{c}}})_j$ for all $j \not = i$, we can choose $\varepsilon > 0$ sufficiently small such that $\hat{c}_j = x_j(\varepsilon ) < (D {\textbf{x}}(\varepsilon ))_j$ for all $j \not = i$ as well, and $0 < x_i(\varepsilon )$. We then have $x_i(\varepsilon ) < \hat{c}_i$, but $(D {\textbf{x}}(\varepsilon ))_i = (D \hat{{\textbf{c}}})_i$, so also $x_i(\varepsilon ) < (D \textbf{x}(\varepsilon ))_i$. Hence ${\textbf{x}}(\varepsilon )$ satisfies ${\textbf{0}}< {\textbf{x}}(\varepsilon ) < D {\textbf{x}}(\varepsilon )$ and $\hat{{\textbf{c}}} \le D {\textbf{x}}(\varepsilon )$. By the above result, we can choose a continuation contribution sequence $({\textbf{c}}^*(t))_t$ such that $\hat{{\textbf{c}}}^*= \textbf{x}(\varepsilon )$. Now consider the sequence $(\hat{{\textbf{c}}}, \delta ^{-1} \bar{{\textbf{c}}}^*(0), \delta ^{-1} \bar{{\textbf{c}}}^*(1), \delta ^{-1} \bar{{\textbf{c}}}^*(2), \dots )$. By definition, $\bar{\textbf{c}}^*(0) = {\textbf{x}}(\varepsilon )$, and we have

$$\begin{aligned} \delta ( \delta ^{-1} {\textbf{x}}(\varepsilon ) ) \le \hat{{\textbf{c}}} \le \delta D ( \delta ^{-1} {\textbf{x}}(\varepsilon ) ), \end{aligned}$$

so by Theorem 1, the sequence we constructed is a sustainable continuation contribution sequence . It begins with $\hat{{\textbf{c}}}$, so the statement of the Lemma holds for $\hat{{\textbf{c}}}$.

Now, the only case that remains is $\hat{{\textbf{c}}} \le _1D \hat{\textbf{c}}$ and ${\textbf{0}} \not < \hat{{\textbf{c}}} \not = {\textbf{0}}$. In the definition of the public goods game, we imposed the conditions $1 < r_i$ and $r_i < n$ for all *i*. We used $r_i < n$ in the proofs about sustainability (it is necessary for *D* being well defined and positive), but $1 < r_i$ was only used to prove statements about maximal welfare. So we can consider games that only satisfy ${\textbf{r}} > {\textbf{0}}$ instead of ${\textbf{r}} > {\textbf{1}}$, and the same results about sustainabililty will apply, including the ones from this proof.

Let $\hat{{\textbf{c}}} \not = {\textbf{0}}$ be any total contribution vector satisfying $\hat{{\textbf{c}}} \le _1D \hat{{\textbf{c}}}$. Let $n'$ be the number of non-zero components of $\hat{{\textbf{c}}}$. W.l.o.g. let these be the first $n'$ components of $\hat{{\textbf{c}}}$. Necessarily $n' > 1$. Let $\hat{{\textbf{c}}}'$ be the first $n'$ components of $\hat{{\textbf{c}}}$, and let ${\textbf{r}}'$ be the $n'$-vector defined by $r'_i = n' n^{-1} r_i$ for all $1 \le i \le n'$. Consider the game $\Gamma _{\!\textrm{B}}({\textbf{r}}', \delta )$ and its zero-diagonal productivity matrix $D'$. We have $D'_{ij} = D_{ij}$ for all $1 \le i, j \le n'$. Since $\hat{{\textbf{c}}}$ satisfies $\hat{\textbf{c}} \le _1D \hat{{\textbf{c}}}$, consequently $\hat{{\textbf{c}}}'$ also satisfies $\hat{{\textbf{c}}}' \le _1D' \hat{{\textbf{c}}}'$. But $\hat{{\textbf{c}}}'$ additionally satisfies ${\textbf{0}} < \hat{{\textbf{c}}}'$. So by the case handled above, there is a sustainable continuation contribution sequence $({\textbf{c}}'(t))_t$ starting with $\hat{{\textbf{c}}}'$. Let $({\textbf{c}}(t))_t$ be the *n*-player sequence with $c'_i(t) = c_i(t)$ for all $i \le n'$ and all *t*, and $c'_i(t) = 0$ for all $i > n'$ and all *t*. Using Eq. 5, we can see that sustainability of $(\textbf{c}(t))_t$ follows trivially from sustainability of $({\textbf{c}}'(t))_t$. So we have found a continuation contribution sequence starting with $\hat{{\textbf{c}}}$.

We have thus shown in the most general case that if some $\hat{\textbf{c}} \not = {\textbf{0}}$ satisfies $\hat{{\textbf{c}}} \le _1D \hat{{\textbf{c}}}$, then $\hat{{\textbf{c}}}$ is sustainable. Together with the converse, which we already showed, this completes the proof.

$$\square $$

### A.2 Proof of Theorem 8

#### Proof of Theorem 8

Theorem 5 says that a total contribution vector of $\hat{{\textbf{c}}}$ is sustainable exactly if

$$\begin{aligned} \hat{{\textbf{c}}} \le _1D \hat{{\textbf{c}}}. \end{aligned}$$

Corollary 3 says that constant contributions of $\hat{{\textbf{c}}}$ are sustainable exactly if

$$\begin{aligned} \hat{\textbf{c}} \le \delta D \hat{{\textbf{c}}}. \end{aligned}$$

Let ${\mathcal {F}}$ be the region defined by $\hat{{\textbf{c}}} \le _1D \hat{{\textbf{c}}}$. Let

$$\begin{aligned} E_{\sup } :=\sup _{\hat{{\textbf{c}}} \in {\mathcal {F}} \setminus \{ \textbf{0} \}} E(\hat{{\textbf{c}}}) = \sup _{\hat{{\textbf{c}}} \in \overline{{\mathcal {F}}} \setminus \{ {\textbf{0}} \}} E(\hat{{\textbf{c}}}), \end{aligned}$$

where $\overline{{\mathcal {F}}}$ is the closure of ${\mathcal {F}}$, which is given by $\hat{{\textbf{c}}} \le D \hat{{\textbf{c}}}$. Firstly, note that $E_{\sup }$ is well defined, since $E(\hat{{\textbf{c}}})$ is bounded above by $\max _i r_i$. We observe that $E(\hat{{\textbf{c}}})$ attains a maximum on $\overline{{\mathcal {F}}} \setminus \{ {\textbf{0}} \}$: We have

$$\begin{aligned} \{ E(\hat{{\textbf{c}}}) {|}\hat{{\textbf{c}}} \in \overline{{\mathcal {F}}} \setminus \{ {\textbf{0}} \} \} = \{ E( \hat{{\textbf{c}}} / \Vert \hat{{\textbf{c}}}\Vert ) {|}\hat{{\textbf{c}}} \in \overline{{\mathcal {F}}} \setminus \{ \textbf{0 }\} \} = \{ E(\hat{{\textbf{c}}}) {|}\hat{{\textbf{c}}} \in \overline{{\mathcal {F}}} \cap S^{n-1} \}, \end{aligned}$$

where $S^{n-1} = \{ {\textbf{x}} \in \mathbb {R}^n {|}\, \Vert {\textbf{x}} \Vert = 1 \}$. Since $\overline{{\mathcal {F}}}$ is closed, $\overline{{\mathcal {F}}} \cap S^{n-1}$ is a compact set. So we can write

$$\begin{aligned} E_{\sup } = \max _{\hat{{\textbf{c}}} \in \overline{{\mathcal {F}}} \setminus \{ {\textbf{0}} \}} E(\hat{{\textbf{c}}}). \end{aligned}$$

Now we are ready to prove the statement of the theorem. But instead of showing that a sustainable time-dependent contribution sequence that is more resource-efficient than all constant contribution sequences exists exactly if

$$\begin{aligned} (\delta (m-1) + 1) r_{\max } < n, \end{aligned}$$

as stated in the theorem, we will show the following equivalent statement: A constant contribution sequence with resource efficiency $E_{\sup }$ exists exactly if

A3 $$\begin{aligned} n \le (\delta (m-1) + 1) r_{\max }. \end{aligned}$$

The following statements are equivalent for $\hat{{\textbf{c}}} \in {\mathbb {R}}^n$:

A4 $$\begin{aligned} \hat{{\textbf{c}}}&\in \overline{{\mathcal {F}}} \nonumber \\ \hat{{\textbf{c}}}&\le D \, \hat{{\textbf{c}}} \nonumber \\ \forall i \quad \hat{c}_i&\le \sum _{j\not =i} \frac{r_j}{n-r_i} \hat{c}_j \end{aligned}$$

A5 $$\begin{aligned} \forall i \quad n \hat{c}_i&\le \sum _j r_j \hat{c}_j . \end{aligned}$$

Take any $\hat{{\textbf{c}}} \in \overline{{\mathcal {F}}} {\setminus } \{ \textbf{0 }\}$ such that $E(\hat{{\textbf{c}}}) = E_{\sup }$ that is sustainable with a constant contribution sequence , which we assume exists. This $\hat{{\textbf{c}}}$ maximises $E(\hat{{\textbf{c}}})$ over all $\hat{{\textbf{c}}} \not = {\textbf{0}}$ satisfying the above inequality (A5). This implies the following statement ($*$): There are no $i_1, i_2$ such that $r_{i_1} > r_{i_2}$ and $n \hat{c}_{i_1} < \sum _j r_j \hat{c}_j$ and $\hat{c}_{i_2} > 0$. Otherwise, we could increase $\hat{c}_{i_1}$ and decrease $\hat{c}_{i_2}$ by equal amounts to increase $E(\hat{{\textbf{c}}})$ while also staying within $\overline{{\mathcal {F}}} {\setminus } \{ {\textbf{0}} \}$.

Since $\hat{{\textbf{c}}}$ is sustainable with a constant contribution sequence , we have $\hat{{\textbf{c}}} \le \delta D \hat{{\textbf{c}}}$, which is equivalent to

A6 $$\begin{aligned} \forall i \quad \hat{c}_i&\le \delta \sum _{j\not =i} \frac{r_j}{n-r_i} \hat{c}_j . \end{aligned}$$

At least two $\hat{c}_i$ must be strictly positive, so $\sum _{j\not =i} \frac{r_j}{n-r_i} \hat{c}_j > 0$ for every *i*. Since $\delta < 1$, (A6) implies

$$\begin{aligned} \forall i \quad \hat{c}_i < \sum _{j\not =i} \frac{r_j}{n-r_i} \hat{c}_j. \end{aligned}$$

So the statement ($*$) simplifies to: There are no $i_1, i_2$ such that $r_{i_1} > r_{i_2}$ and $\hat{c}_{i_2} > 0$. This means that for any *i*, if $\hat{c}_i > 0$, then $r_i = r_{\max }$.

From (A6), we get

A7 $$\begin{aligned} \forall i \quad (n - (1-\delta ) r_i) \hat{c}_i&\le \delta \sum _j r_j \hat{c}_j , \end{aligned}$$

where

$$\begin{aligned} \sum _j r_j \hat{c}_j = r_{\max } \sum _j \hat{c}_j. \end{aligned}$$

Exactly *m* components of $\hat{{\textbf{c}}}$ are non-zero. So choose some *i* such that $\hat{c}_i > 0$ and

$$\begin{aligned} \hat{c}_i \ge \frac{1}{m} \sum _j \hat{c}_j. \end{aligned}$$

Inserting into (A7), we get

$$\begin{aligned} (n - (1-\delta ) r_{\max }) \frac{1}{m} \sum _j \hat{c}_j \le \delta r_{\max } \sum _j \hat{c}_j. \end{aligned}$$

The $\hat{c}_j$ sum to 1. We multiply with *m* on both sides and get

$$\begin{aligned} n - (1-\delta ) r_{\max } \le \delta r_{\max } m. \end{aligned}$$

By simple rearrangement, this is equivalent to (A3). So (A3) being false is a necessary condition for the existence of a constant contribution sequence with resource efficiency $E_{\sup }$, which was our only assumption.

Now instead assume conversely that (A3) holds. Let ${\hat{c}}_i = 1$ for all *i* such that $r_i = r_{\max }$, and let ${\hat{c}}_i = 0$ for all other *i*. We can check easily from the definitions that $\hat{{\textbf{c}}}$ satisfies $\hat{{\textbf{c}}} \le \delta D \hat{{\textbf{c}}}$, so it is sustainable with a constant contribution sequence . The resource efficiency of $\hat{{\textbf{c}}}$ is $E(\hat{{\textbf{c}}}) = r_{\max }$, so it is maximal. Therefore, (A3) is also a sufficient condition, and hence an exact condition, for the existence of a constant contribution sequence with resource efficiency $E_{\sup }$. $\square$

### A.3 Proof of Theorem 12

#### Proof of Theorem 12

We will show that if ${\textbf{e}} \le \delta D {\textbf{e}}$, then $W_{\sup }^{\textrm{s}}({\textbf{e}}) = W_{\sup }({\textbf{e}})$, and if ${\textbf{e}} \not \le \delta D {\textbf{e}}$, then $W_{\sup }^{\textrm{s}}({\textbf{e}}) > W_{\sup }({\textbf{e}})$.

Firstly, if ${\textbf{e}} \le \delta D {\textbf{e}}$, then by Corollary 3, the constant contribution sequence $({\textbf{c}}(t))_t = ({\textbf{e}})_t$ is sustainable. So the total contribution vector $\hat{{\textbf{c}}} = {\textbf{e}}$ is sustainable without saving. Since ${\textbf{e}}$ is an upper bound on the total contribution vector , ${\textbf{r}}^\intercal {\textbf{e}}$ is also an upper bound on welfare in general, and we have $W_{\sup }^{\textrm{s}}({\textbf{e}}) = W_{\sup }({\textbf{e}}) = {\textbf{r}}^\intercal {\textbf{e}}$.

Now, assume that ${\textbf{e}} \not \le \delta D {\textbf{e}}$. By Proposition 11, there is always a total contribution vector $\hat{\mathbf {{c}}}$ that satisfies $W(\hat{{\textbf{c}}}) = W_{\sup }({\textbf{e}})$ and is sustainable with a constant contribution sequence in $\Gamma _{\!\textrm{E}}(\textbf{r}, {\textbf{e}}, \delta )$, i.e. without saving. Take such a $\hat{{\textbf{c}}}$. By Corollary 3, we have $\hat{{\textbf{c}}} \le \delta D \hat{{\textbf{c}}}$. So $\hat{{\textbf{c}}} \not = {\textbf{e}}$, meaning there is some *i* such that $\hat{c}_i < e_i$. Fix such an *i*.

Define $\hat{{\textbf{c}}}(\varepsilon ) = \hat{{\textbf{c}}} + \varepsilon ({\textbf{e}} - \hat{{\textbf{c}}})$ for $\varepsilon \ge 0$. Clearly $\hat{\textbf{c}}(\varepsilon ) \rightarrow \hat{{\textbf{c}}}$ as $\varepsilon \rightarrow 0$. Since $\hat{{\textbf{c}}} \le \delta D \hat{{\textbf{c}}}$, since $D \hat{{\textbf{c}}}$ is a positive vector, and since $\delta < 1$, we have $\hat{{\textbf{c}}} < D \hat{{\textbf{c}}}$. So we can choose $\varepsilon > 0$ sufficiently small such that $\hat{{\textbf{c}}}(\varepsilon ) < D \hat{{\textbf{c}}}(\varepsilon )$ as well. By Theorem 5, $\hat{{\textbf{c}}}(\varepsilon )$ is thus a sustainable total contribution vector in $\Gamma _{\!\textrm{S}}({\textbf{r}}, {\textbf{e}}, \delta )$. But $\hat{\textbf{c}}(\varepsilon ) \ge \hat{{\textbf{c}}}$ and $\hat{c}(\varepsilon )_i > \hat{c}_i$. So $W(\hat{{\textbf{c}}}(\varepsilon )) > W(\hat{{\textbf{c}}})$. Consequently, $W_{\sup }^{\textrm{s}}({\textbf{e}}) > W_{\sup }({\textbf{e}})$. $\square$

### A.4 Proof of Theorem 13

#### Proposition 14

Take any game $\Gamma _{\!\textrm{B}}({\textbf{r}}, \delta )$ that allows for non-defection. Then for any $\hat{{\textbf{c}}} \in {\mathbb {R}}^n_{\ge 0}$ satisfying $\sum _{i=1}^n \hat{c}_i \le 1$, the following are equivalent:

1. A total contribution vector of $\hat{{\textbf{c}}}$ is sustainable in the game $\Gamma _{\!\textrm{B}}({\textbf{r}}, \delta )$.
2. There exists an endowment distribution $\textbf{e}$ such that a total contribution vector of $\hat{{\textbf{c}}}$ is sustainable in the game $\Gamma _{\!\textrm{S}}({\textbf{r}}, {\textbf{e}}, \delta )$.

In a game $\Gamma _{\!\textrm{S}}({\textbf{r}}, {\textbf{e}}, \delta )$, a contribution sequence $(\textbf{c}(t))_t$ is called sustainable if there is a play that results in contribution sequence $({\textbf{c}}(t))_t$.

It is easy to check that a contribution sequence $({\textbf{c}}(t))_t$ is sustainable in $\Gamma _{\!\textrm{S}}({\textbf{r}}, {\textbf{e}}, \delta )$ if and only if

A8 $$\begin{aligned} \hat{{\textbf{c}}} \le \delta ^t {\textbf{e}} + \delta ^{t+1} \bar{{\textbf{c}}}(t+1) \end{aligned}$$

for all $t \ge 0$. Inequality A8 simply states that every player *i* has enough resources in every round *t* in order to make a contribution of $c_i(t)$ as long as they never consume any of their available resources privately.

In the game $\Gamma _{\!\textrm{B}}({\textbf{r}}, \delta )$, the Grim strategy profile $G(({\textbf{c}}(t))_t)$ for a contribution sequence $({\textbf{c}}(t))_t$ is the pure strategy profile $G(({\textbf{c}}(t))_t) = (\sigma _i)_i$ defined as follows: In each round *t* and for each *i* the strategy $\sigma _i$ contributes $c_i(t)$ if all players have so far also played according to $({\textbf{c}}(t))_t$, but otherwise contributes 0.

In the base model, a contribution sequence $({\textbf{c}}(t))_t$ is sustainable in a given game if and only if its associated Grim strategy profile $G((\textbf{c}(t))_t)$ is a SPE of the game [23].

In the game $\Gamma _{\!\textrm{B}}({\textbf{r}}, {\textbf{e}}, \delta )$, the Grim strategy profile $G^\textrm{s}(({\textbf{c}}(t))_t)$ for a contribution sequence $(\textbf{c}(t))_t$ is defined as follows.

First, we recursively construct a deposit sequence $({\textbf{d}}(t))_t$. For each *t* and each *i*, let $d_i(t)$ be the minimal *d* such that there is a play that results in contribution sequence $({\textbf{c}}(t))_t$ and an initial deposit sequence for player *i* of $d_i(0)$, ..., $d_i(t-1)$, *d*. We can check that a minimum is indeed attained. Then there also exists a play that results in contribution sequence $({\textbf{c}}(t))_t$ and deposit sequence $({\textbf{d}}(t))_t$. This also uniquely determines the private consumption sequence $({\textbf{p}}(t))_t$.

Now the Grim strategy $G^\textrm{s}(({\textbf{c}}(t))_t)_i$ for player *i* plays as follows. In each round *t*, if all players have so far played according to $G^\textrm{s}(({\textbf{c}}(t))_t)$, then player *i* contributes $c_i(t)$, privately consumes $p_i(t)$, and deposits $d_i(t)$. Otherwise, player *i* contributes 0, deposits 0, and privately consumes the entire available amount.

We see that in both models, the Grim strategy profile $G((\textbf{c}(t))_t)$ or $G^\textrm{s}(({\textbf{c}}(t))_t)$, respectively, produces the contribution sequence $({\textbf{c}}(t))_t$. Hence, in both models, there is a bijective correspondence between sustainable contribution sequences and Grim strategies.

#### Proof of Proposition 14

Take any $\hat{{\textbf{c}}} \in {\mathbb {R}}^n_{\ge 0}$ satisfying $\sum _{i=1}^n \hat{c}_i \le 1$.

Assume that statement 1. of Proposition 14 holds. By Theorem 5, choose a sustainable contribution sequence $({\textbf{c}}(t))_t$ such that $\hat{{\textbf{c}}} = \bar{\textbf{c}}(0)$ and $\hat{{\textbf{c}}} \le \bar{{\textbf{c}}}(t)$ for all *t*. We know that $G(({\textbf{c}}(t))_t)$ is a SPE of the game $\Gamma _{\!\textrm{B}}({\textbf{r}}, \delta )$.

Set ${\textbf{e}} = \hat{{\textbf{c}}}$. We want to show that in the game $\Gamma _{\!\textrm{S}}({\textbf{r}}, {\textbf{e}}, \delta )$, the Grim strategy profile $G^\textrm{s}(({\textbf{c}}(t))_t)$ is a SPE, which is sufficient to show that statement 2. holds. Let $({\textbf{d}}(t))_t$ be the deposit sequence produced by $G^\textrm{s}(({\textbf{c}}(t))_t)$. So consider a strategy profile where all players play according to $G^\textrm{s}(({\textbf{c}}(t))_t)$ with the execption of one mutant, *i*, who plays according to a general strategy $\sigma$. Let $(\textbf{c}'(t))_t$ be the contribution sequence , $({\textbf{p}}'(t))_t$ the private consumption sequence, and $({\textbf{d}}'(t))_t$ the deposit sequence produced by this strategy profile. Let ${\pi ^\textrm{s}}'_i$ be the payoff of player *i*, and let $\pi ^\textrm{s}_i$ be the payoff of player *i* if player *i* were also playing according to $G^\textrm{s}((\textbf{c}(t))_t)$. We need to show that ${\pi ^\textrm{s}}'_i \le \pi ^\textrm{s}_i$.

Now, in the game $\Gamma _{\!\textrm{B}}({\textbf{r}}, \delta )$, consider a strategy profile where all players play according to $G((\textbf{c}(t))_t)$ with the execption of one mutant, *i*, who unconditionally plays the contribution sequence $(c'_i(t))_t$. Clearly, this will also result in the same contribution sequence $({\textbf{c}}'(t))_t$. Let $\pi '_i$ be the payoff of player *i* in this strategy profile, and $\pi _i$ the payoff of player *i* if player *i* were also playing according to $G(({\textbf{c}}(t))_t)$.

We have $\sum _{t=0}^\infty \delta ^t p'_i(t) \le \sum _{t=0}^\infty \delta ^t d'_i(t)$, so ${\pi ^\textrm{s}}'_i \le \pi _i'$. By the minimality of the deposit sequence $({\textbf{d}}(t))_t$ according to the definition of Grim strategies in the savings model, we have $\sum _{t=0}^\infty \delta ^t p_i(t) = \sum _{t=0}^\infty \delta ^t d_i(t)$. So $\pi ^\textrm{s}_i = \pi _i$. Since $G(({\textbf{c}}(t))_t)$ is a SPE of the game $\Gamma _{\!\textrm{B}}({\textbf{r}}, \delta )$, we have $\pi _i' \le \pi _i$. We therefore have that ${\pi ^\textrm{s}}'_i \le \pi ^\textrm{s}_i$.

Now instead assume that statement 2. holds. Fix any ${\textbf{e}}$ and any SPE of the game $\Gamma _{\!\textrm{S}}({\textbf{r}}, {\textbf{e}}, \delta )$ which produces some contribution sequence $({\textbf{c}}(t))_t$, sequence of deposits $({\textbf{d}}(t))_t$ and the total contribution vector $\hat{{\textbf{c}}}$. By the definition of an SPE, no player can at any time profit from switching to privately consuming the entire amount that they have at their disposal in each round for the rest of the game.

Take any player *i* and any time *t*. The continuation payoff of player *i* at time *t* is ordinarily

$$\begin{aligned} {\bar{\pi }}_i(t)&= (1-\delta ) \sum _{\tau = t}^\infty \delta ^{\tau -t} \pi _i(\tau ) \\&= (1-\delta ) \sum _{\tau = t}^\infty \delta ^{\tau -t} ( p_i(\tau ) + n^{-1} {\textbf{r}}^\intercal {\textbf{c}}(\tau )) \\&= (1-\delta ) \sum _{\tau = t}^\infty \delta ^{\tau -t} ( e_i + s_i(\tau ) - c_i(\tau ) - d_i(\tau ) + n^{-1} {\textbf{r}}^\intercal \textbf{c}(\tau )) \\&= e_i + (1-\delta ) s_i(t) - {\bar{c}}_i(t) + n^{-1} {\textbf{r}}^\intercal \bar{{\textbf{c}}}(t) , \end{aligned}$$

where the last equality holds because $\delta s_i(\tau + 1) = d_i(\tau )$.

If the player switched at time *t* to $c'_i(\tau ) = 0$ and $p'_i(\tau ) = e_i + s'_i(\tau )$ for all $\tau \ge t$, then the continuation payoff would be

$$\begin{aligned} (1-\delta ) ^{-1} {\bar{\pi }}_i'(t)&= \pi _i'(t) + \sum _{\tau = t+1}^\infty \delta ^{\tau -t} \pi _i(\tau ) \\&= p'_i(t) + n^{-1} {\textbf{r}}^\intercal {\textbf{c}}'(t) + \sum _{\tau = t+1}^\infty \delta ^{\tau -t} ( p_i'(\tau ) + n^{-1} {\textbf{r}}^\intercal {\textbf{c}}'(\tau ) ) \\&= e_i + s_i(t) + n^{-1} ({\textbf{r}}^\intercal {\textbf{c}}(t) - r_i c_i(t)) + \sum _{\tau = t+1}^\infty \delta ^{\tau -t} ( e_i + n^{-1} \textbf{r}^\intercal {\textbf{c}}'(\tau ) ) \\&= (1-\delta ) ^{-1} e_i + s_i(t) + n^{-1} ({\textbf{r}}^\intercal {\textbf{c}}(t) - r_i c_i(t)) + \sum _{\tau = t+1}^\infty \delta ^{\tau -t} n^{-1} {\textbf{r}}^\intercal {\textbf{c}}'(\tau ) \\&\ge (1-\delta ) ^{-1} e_i + s_i(t) + n^{-1} ({\textbf{r}}^\intercal \textbf{c}(t) - r_i c_i(t)) . \end{aligned}$$

So ${\bar{\pi }}_i'(t) \le {\bar{\pi }}_i(t)$ implies the following equivalent statements:

$$\begin{aligned} e_i + (1-\delta ) s_i(t) + (1-\delta ) n^{-1} ({\textbf{r}}^\intercal {\textbf{c}}(t) - r_i c_i(t))&\le e_i + (1-\delta ) s_i(t) - {\bar{c}}_i(t) + n^{-1} {\textbf{r}}^\intercal \bar{{\textbf{c}}}(t) \\ n {\bar{c}}_i(t) + (1-\delta ) \sum _{j \not = i} r_j c_j(t)&\le \sum _{j} r_j \bar{c}_j(t) \\ n {\bar{c}}_i(t) + (1-\delta ) \sum _{j \not = i} r_j c_j(t)&\le r_i \bar{c}_i(t) + (1-\delta ) \sum _{j \not = i} r_j c_j(t) + \delta \\ \sum _{j \not = i} r_j \bar{c}_j(t+1) \\ (n-r_i) {\bar{c}}_i(t)&\le \delta \sum _{j \not = i} r_j \bar{c}_j(t+1) \\ {\bar{c}}_i(t)&\le (\delta D \bar{{\textbf{c}}}(t+1))_i . \end{aligned}$$

So the continuation contribution sequence $(\bar{{\textbf{c}}}(t))_t$ satisfies $\bar{{\textbf{c}}}(t) \le \delta D \bar{{\textbf{c}}}(t+1)$ for all *t*. By Theorem 1 therefore, it is sustainable in the game $\Gamma _{\!\textrm{B}}({\textbf{r}}, \delta )$. So the total contribution vector $\hat{{\textbf{c}}}$ is also sustainable in that setting.

So the two statements are equivalent. $\square$

#### Proof of Theorem 13

Let $(r, \delta )$ allow for non-defection.

Write ${\mathcal {W}}^{\textrm{s}}$ for the set of welfare values attainable with sustainable contribution sequences in the savings model. Write ${\mathcal {E}}$ for the set of resource-efficiencies of non-defective SPEs in the base model. We can show that $\sup {\mathcal {W}}^{\textrm{s}} = \sup {\mathcal {E}} + 1$. First, assume that $W \in {\mathcal {W}}^{\textrm{s}}$. Then, in the savings model, there is ${\textbf{e}}$ and a sustainable $\hat{{\textbf{c}}}$ such that $W = W_{{\textbf{e}}}(\hat{{\textbf{c}}})$. We have

$$\begin{aligned} W = W_{{\textbf{e}}}(\hat{{\textbf{c}}}) = \sum _i \hat{\pi }_i = \sum _i (\hat{\pi }_i - e_i) + 1 \le \frac{\sum _i (\hat{\pi }_i - e_i)}{\sum _i \hat{{\textbf{c}}}_i} + 1 = E(\hat{{\textbf{c}}}) + 1. \end{aligned}$$

The value of $E(\hat{{\textbf{c}}})$ is the same in both models. So $W \le \sup {\mathcal {E}} + 1$.

Now take some $E \in {\mathcal {E}}$. In the base model, take a sustainable, non-defective $\hat{{\textbf{c}}}$ such that $E = E(\hat{\textbf{c}})$. By Corollary 2 and linearity of $E(\hat{\textbf{c}})$, we can assume $\sum _i \hat{c}_i = 1$. By Proposition 14, take ${\textbf{e}}$ such that $\hat{{\textbf{c}}}$ is sustainable in the savings model. Now,

$$\begin{aligned} E + 1 = E(\hat{{\textbf{c}}}) + 1 = \frac{\sum _i (\hat{\pi }_i - e_i)}{\sum _i \hat{{\textbf{c}}}_i} + 1 = \sum _i \hat{\pi }_i = W_{\textbf{e}}(\hat{{\textbf{c}}}). \end{aligned}$$

So $E + 1 \le \sup {\mathcal {W}}^{\textrm{s}}$. We therefore have $\sup {\mathcal {W}}^{\textrm{s}} = \sup {\mathcal {E}} + 1$. We write this as $W_{\sup }^{\textrm{s}} = E_{\sup } + 1$.

Write ${\mathcal {W}}$ for the set of welfare values that can be achieved with any ${\textbf{e}}$ in the endowment model. We already defined $W_{\sup } = \sup {\mathcal {W}}$. Write ${\mathcal {E}}^{\textrm{c}}$ for the set of resource-efficiencies of non-defective SPEs in the base model that are achieved with constant contribution sequences , and $E_{\sup }^{\textrm{c}}$ for its supremum. We now show that $W_{\sup } = E_{\sup }^{\textrm{c}} + 1$.

[23] show (their Lemma 3) that $W_{\sup }$ is attained, and that it is attained with a sustainable constant contribution sequence . Naturally, this requires that $\hat{{\textbf{c}}} = {\textbf{e}}$, that is, full contribution. But at full contribution, $W_{\textbf{e}}(\hat{{\textbf{c}}}) = E(\hat{{\textbf{c}}}) + 1$. So $W_{\sup } \le E_{\sup }^{\textrm{c}} + 1$.

Now take some $E \in {\mathcal {E}}^{\textrm{c}}$. In the base model, take a non-defective $\hat{{\textbf{c}}}$ such that $E = E(\hat{{\textbf{c}}})$, which is sustainable with a constant contribution sequence . Set ${\textbf{e}} = \hat{\textbf{c}}$. Since the constant sequence $({\textbf{e}})_t$ is sustainable in the base model, $\hat{{\textbf{c}}}$ is also sustainable with the endowment constraint ${\textbf{e}}$. Again, we have $E + 1 = W_{{\textbf{e}}}(\hat{{\textbf{c}}})$. So $E + 1 \le W_{\sup }$. We therefore have $W_{\sup } = E_{\sup }^{\textrm{c}} + 1$.

Making use of both of the identities that we derived, we have that $W_{\sup }^{\textrm{s}} > W_{\sup }$ is equivalent to $E_{\sup } > E_{\sup }^{\textrm{c}}$. The statement now follows from Theorem 8. $\square$

## Declarations

### Computer Code Availability

The example data shown in Fig. 3 were generated with GNU Octave 8.4 and Python 3.12. The computer code used is published in [18].
